# Ainsliadimer A selectively inhibits IKKα/β by covalently binding a conserved cysteine

**DOI:** 10.1038/ncomms7522

**Published:** 2015-03-27

**Authors:** Ting Dong, Chao Li, Xing Wang, Longyang Dian, Xiuguo Zhang, Lin Li, She Chen, Ran Cao, Li Li, Niu Huang, Sudan He, Xiaoguang Lei

**Affiliations:** 1 Graduate School of Peking Union Medical College and Chinese Academy of Medical Sciences, Beijing 100730, China; 2 National Institute of Biological Sciences (NIBS), Beijing 102206, China; 3 Beijing National Laboratory for Molecular Sciences, Department of Chemical Biology, College of Chemistry and Molecular Engineering, Synthetic and Functional Biomolecules Center, and Peking-Tsinghua Center for Life Sciences, Peking University, Beijing 100871, China; 4 Cyrus Tang Hematology Center, Jiangsu Institute of Hematology, First Affiliated Hospital, and Collaborative Innovation Center of Hematology, Soochow University, Suzhou 215123, China

## Abstract

Aberrant activation of NF-κB is associated with the development of cancer and autoimmune and inflammatory diseases. IKKs are well recognized as key regulators in the NF-κB pathway and therefore represent attractive targets for intervention with small molecule inhibitors. Herein, we report that a complex natural product ainsliadimer A is a potent inhibitor of the NF-κB pathway. Ainsliadimer A selectively binds to the conserved cysteine 46 residue of IKKα/β and suppresses their activities through an allosteric effect, leading to the inhibition of both canonical and non-canonical NF-κB pathways. Remarkably, ainsliadimer A induces cell death of various cancer cells and represses *in vivo* tumour growth and endotoxin-mediated inflammatory responses. Ainsliadimer A is thus a natural product targeting the cysteine 46 of IKKα/β to block NF-κB signalling. Therefore, it has great potential for use in the development of anticancer and anti-inflammatory therapies.

## Abstract (web summary)

IKK is a key inducer of NF-κB, and has been targeted by several small molecule drugs. Here the authors show that a natural product from a Chinese medical herb inhibits NF-κB via covalent binding to a unique conserved region of IKK, and efficiently inhibits tumour growth and sepsis in mice.

## Graphical abstract

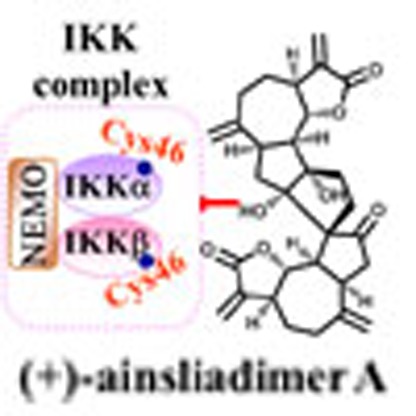

The evolutionarily conserved nuclear factor-κB (NF-κB) signalling pathway plays key roles in inflammatory and immune responses and in cell survival by regulating the transcription of numerous target genes^1^,^2^,^3^,^4^. The NF-κB family of transcription factors consists of five members, including p50, p52, p65 (RelA), c-Rel and RelB, which form various dimeric complexes. The NF-κB dimers are normally sequestered in the cytoplasm by association with a member of the IκB inhibitory family (for example, IκBα, IκBβ, IκBε) or with the precursor proteins p100 and p105. NF-κB activation typically occurs by nuclear translocation of NF-κB dimers following inducible degradation of IκB, or processing of precursor proteins in response to a variety of stimuli, including the presence of cytokines like TNF-α or IL-1, growth factors, microbial infection and/or chemotherapeutic agents.

Canonical NF-κB activation depends on the degradation of IκB, which is rapidly phosphorylated by an active IκB kinase (IKK) complex. This complex is composed of IKKα and IKKβ catalytic subunits and a regulatory subunit, IKKγ/NEMO (NF-κB essential modulator)^5^. IKKβ is the major subunit responsible for phosphorylation of IκB proteins. For example, IκBα is phosphorylated at Ser-32 and Ser-36 (ref. 6), whereas IκBβ is phosphorylated at Ser-19 and Ser-23 (ref. 7). Phosphorylated IκB subsequently undergoes proteasome-mediated degradation, thereby liberating free NF-κB dimers to translocate to the nucleus that can then promote gene transcription^8^. In addition, an alternative pathway designated as the ‘non-canonical NF-κB pathway’ relies on the inducible processing of p100 (ref. 9). This pathway mainly activates IKKα, which in turn phosphorylates p100 to trigger its proteolytic processing to p52, leading finally to nuclear translocation of p52-containing NF-κB dimers.

Aberrant activation of the NF-κB signalling pathway is known to be involved in a variety of human diseases including cancer, autoimmune diseases and chronic inflammatory diseases^2^,^10^,^11^. The NF-κB pathway is important for cancer development and progression, in that it regulates a wide variety of target genes involved in cell proliferation, cell survival, invasion, angiogenesis and metastasis^12^. Continuous activation of NF-κB is a common feature in the majority of human cancers, including both solid and haematopoietic malignancies^13^. Activated NF-κB induces expression of anti-apoptotic genes, including those of the inhibitor of apoptosis protein family^14^, anti-apoptotic Bcl-2 family^15^,^16^ and cellular FLICE-inhibitory protein^17^, which is associated with increased resistance of cancer cells to chemotherapy. Moreover, IKKβ has been recently shown to phosphorylate BAD, which results in the blocking of BAD-mediated apoptosis^18^. In addition to its critical role in cancer, enhanced NF-κB activity is a hallmark of various autoimmune and inflammatory diseases. Chronic inflammatory conditions have been shown to drive an increased cancer risk. Examples of this include colitis-associated colon cancer and hepatitis-associated liver cancer^19^,^20^. Ample evidence suggests that inhibition of NF-κB activity represses cancer cell survival, *in vivo* tumour growth and inflammatory responses. Therefore, strategies focused on reducing NF-κB activity by specific small molecule inhibitors could offer significant therapeutic value for the treatment of these diseases.

Over the past decade, there has been a concerted effort to identify small molecule inhibitors of IKKβ because of its central role in the canonical NF-κB pathway. Some of the small molecule inhibitors that have been identified in these efforts have exerted promising inhibitory effects in various experimental models of tumour and inflammatory diseases^12^,^21^. However, there is as yet limited clinical experience of the efficacy and safety of such molecules. Therefore, it is of great importance that novel IKKα/β inhibitors with unique binding properties, high efficacy and low toxicity are identified and developed as therapeutic agents to suppress both canonical and non-canonical NF-κB activation. Such efforts also enable the dissection of the mechanisms of IKK regulation of the NF-κB signalling pathway. In the current study, we demonstrate that the natural product ainsliadimer A (**1**) tightly binds IKKα and IKKβ through the conserved cysteine residue 46(C46), leading to the inactivation of both the canonical and the non-canonical NF-κB signalling pathways triggered by multiple stimuli. To our knowledge, ainsliadimer A represents the first small molecule natural product targeting the functional C46 of IKKα/IKKβ. Ainsliadimer A leads to IKKα/IKKβ inactivation through a novel allosteric effect. Moreover, ainsliadimer A powerfully blocks LPS-mediated inflammatory responses and tumour growth *in vivo*. These discoveries not only indicate that ainsliadimer A indeed acts as a potent inhibitor of IKKα/β, but also reveal that ainsliadimer A could be used as a lead compound in the development of new therapeutic agents for the treatment of cancer and inflammatory disorders.

## Results

### Ainsliadimer A inhibits LPS-induced inflammatory cytokines *in vivo*

**Figure 1:**
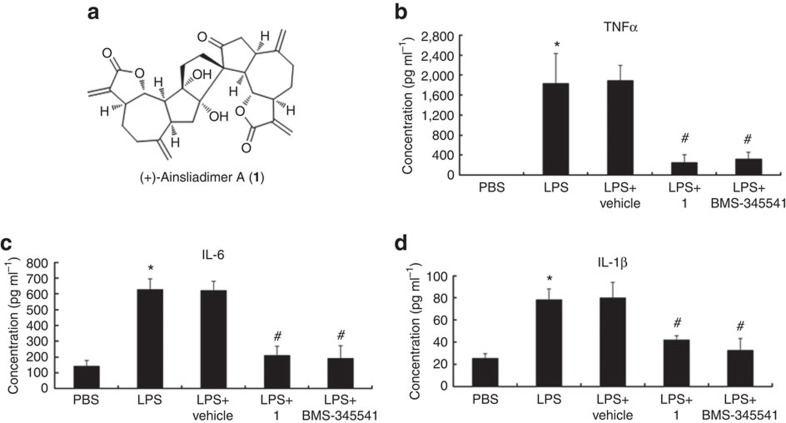
Ainsliadimer A (1) suppresses the production of inflammatory cytokines *in vivo*. Chemical structure of ainsliadimer A (**1**). (**b**–**d**) Effects of ainsliadimer A (**1**) on the production of serum TNF, IL-6 and IL-1β induced by LPS. The mice were administered intravenously with ainsliadimer A (1) (25 mg kg^−1^) (LPS+ainsliadimer A group), BMS-345541(25 mg kg^−1^) (LPS+BMS-345541group), Vehicle (LPS+Vehicle group), or PBS(PBS group) for 1.5 h before intraperitoneal injection of LPS (1.6 mg kg^−1^). Serum concentrations of TNF, IL-6 and IL-1β in the four groups were measured after 3.5 h following LPS injection. Mean±s.e.m. *, *P*< 0.05 versus PBS; #, *P*<0.01 versus LPS. For each group, plasma samples were obtained from at least six mice and were analysed in duplicate. Statistical significance was determined by Student’s *t*-test.

Ainsliadimer A (**1**) is a structurally unique and complex sesquiterpene lactone dimer with an unprecedented carbon skeleton (Fig. 1a) that was originally isolated from *Ainsliaea macrocephala*, a plant that has been used in traditional Chinese medicine for the treatment of various diseases, including angina and rheumatoid arthritis^22^. In our systematic chemical genetic studies using bioactive natural products, we have successfully accomplished the first enantioselective total syntheses of ainsliadimer A (**1**)^23^, as well as that of other related dimeric and trimeric sesquiterpenoids^24^,^25^. Such synthetic efforts have provided us with ample material to extensively investigate both the biological activities and potential therapeutic applications of such compounds. Ainsliadimer A (**1**) has been previously shown to exhibit inhibitory activity against the production of nitric oxide (NO) in a mouse macrophage cell line RAW264.7 stimulated by lipopolysaccharide (LPS)^22^, a major cell wall component of Gram-negative bacteria. As an endotoxin, LPS is known to cause a systemic inflammatory response and acute tissue injury when injected into mice. Thus, we here attempted to examine the effect of ainsliadimer A (**1**) on LPS-triggered inflammatory responses *in vivo*. Mice were pre-injected with or without Vehicle or ainsliadimer A (**1**), followed by administration of PBS or LPS. As shown in Fig. 1b-d, serum inflammatory cytokines including TNF-α, IL-6 and IL-1β were highly induced in the mice challenged with LPS, as compared with the PBS-treated mice, and the upregulation of these cytokines was greatly attenuated in the mice that had been pretreated with ainsliadimer A (**1**) but not in the Vehicle-pretreated mice. As such, it can be concluded that ainsliadimer A (**1**) has great potential as an anti-inflammatory agent *in vivo*. Furthermore, exogenously administered ainsliadimer A (**1**) (25 mg kg^−1^) was shown to cause a significant decrease in NF-κB activation in the spleen after intravenous injection (as compared with Vehicle-treated mice) following LPS challenge (Supplementary Fig. 1).

### Ainsliadimer A acts as a potent inhibitor of NF-κB pathways

**Figure 2:**
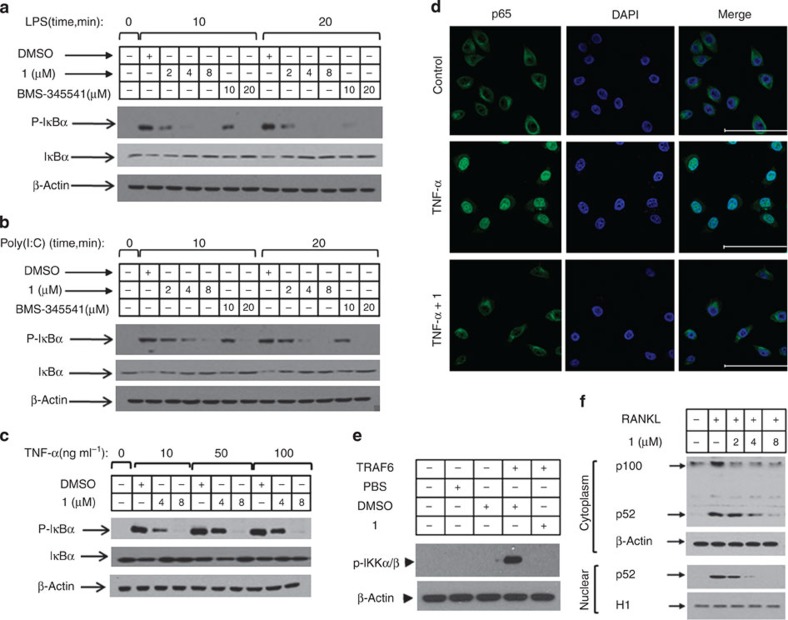
Ainsliadimer A (1) blocks NF-κB activation induced by diverse stimuli in various cells. Raw 264.7 cells (**a**,**b**), and HeLa cells (**c**) were preincubated with the indicated concentrations of ainsliadimer A (**1**) for 1 h before stimulation with 20 ng ml^−1^ LPS (**a**), 20 μg ml^−1^ Poly(I:C) (**b**) or TNF-α (**c**). Cells were harvested at the indicated time points, and total cell lysates were tested by western blot experiments for the occurrence of IκBα phosphorylation. DMSO and BMS-345541 were used as negative and positive controls, respectively. All experiments were repeated at least three times, and similar results were obtained each time. (**d**) Ainsliadimer A (**1**) inhibited TNF-α-induced NF-κB/P65 nuclear translocation. HeLa cells were pretreated with ainsliadimer A (**1**, 8 μM) for 1.5 h and then treated with TNF-α (20 ng ml^−1^) for an additional 0.5 h. DMSO was used as a negative control. After treatment, cells were stained with a primary anti-p65 antibody, and then the nucleus was counterstained with DAPI (blue) and examined using fluorescence microscopy. Scar bar, 100 μm. (**e**) Effects of ainsliadimer A (**1**) on TRAF6-induced phosphorylation of IKKα and IKKβ in cell-free extracts. S100 cell lysates from 293T cells were incubated with a PBS control, recombinant TRAF6, DMSO or ainsliadimer A (**1**), and their action was analysed by immunoblotting using antibodies, as indicated. (**f**) Effects of ainsliadimer A (**1**) on the non-canonical NF-κB pathway induced by RANKL. RAW264.7 cells were preincubated with the indicated concentration of ainsliadimer A (**1**) for 1.5 h, and then stimulated with 150 ng ml^−1^ RANKL for 30 min. The cells were then harvested and the cytoplasmic fractions and nuclear fractions were extracted and subjected to immunoblotting with the indicated antibodies.

The potent anti-inflammatory activity of ainsliadimer A (**1**) prompted us to investigate how it modulates pro-inflammatory cytokine responses. As a well-defined agonist of TLR4 (refs 12, 13), LPS specifically activates TLR4 and subsequently results in NF-κB activation and type I IFN responses. We next evaluated the effect of ainsliadimer A (**1**) on LPS-induced NF-κB activation. Phosphorylation of IκB is a key step in the activation of the canonical NF-κB pathway. As expected, phosphorylated IκBα was detected in RAW264.7 cells after exposure to LPS for 10 or 20 min. Strikingly, pretreatment with 8 μM ainsliadimer A (**1**) could effectively block the phosphorylation of IκBα (Fig. 2a). In comparison with the well-known NF-κB inhibitor BMS-345541 26, which suppressed LPS-induced phosphorylation of IκBα at 20 μM, ainsliadimer A (**1**) exhibited much stronger inhibitory effects against LPS-induced NF-κB activation. Meanwhile, by assessing IFR3 phosphorylation, we found that ainsliadimer A (**1**) had no effect on LPS-induced type I IFN responses, indicating that ainsliadimer A (**1**) specifically targets LPS-trigged NF-κB signalling (Supplementary Fig. 2). Furthermore, ainsliadimer A (**1**) markedly suppressed the phosphorylation of IκBα triggered by synthesized dsRNA poly(I:C), a TLR3 ligand^27^ (Fig. 2b). These results demonstrate that ainsliadimer A (**1**) affects a common signalling component shared in both TLR3- and TLR4-mediated NF-κB signalling. In addition to the TLRs, the engagement of death receptors with corresponding ligands including TNF-α is also known to trigger NF-κB activation. We found that ainsliadimer A (**1**) significantly inhibited TNF-α-induced phosphorylation of IκBα in various cell lines (Fig. 2c and Supplementary Fig. 3). Moreover, addition of ainsliadimer A (**1**) abolished TNF-α-induced nuclear translocation of p65 (Fig. 2d). Taken together, these results establish that ainsliadimer A (**1**) acts as a potent inhibitor of the NF-κB pathway induced by diverse stimuli.

TNF receptor-associated factors (TRAFs) are important intermediates in the NF-κB signalling pathway. For example, TRAF2 and TRAF5 regulate TNF-α receptor-triggered IKK activation, and TRAF6 is required for IL-1-induced IKK activation^2^. The latter pathway can be reconstituted in cell-free extracts by adding recombinant TRAF6. As shown in Fig. 2e, phosphorylation of IKKα/β was detected in S100 cell lysates from 293T cells incubated with recombinant TRAF6. Notably, the addition of ainsliadimer A (**1**) completely abolished TRAF6-induced phosphorylation of IKKα/β, indicating that ainsliadimer A (**1**) disrupts the NF-κB pathway by intervening in the function of the IKK complex or that of upstream activators.

We then tested whether ainsliadimer A (**1**) affected the non-canonical NF-κB pathway, which is activated via a subset of TNFR superfamily members, including the receptor activator for nuclear factor κB (RANK), a B-cell-activating factor belonging to the TNF family receptor (BAFFR) and a lymphotoxinβ-receptor (LTβR)^9^. As shown in Fig. 2f, the processing of p100 to p52 triggered by RANKL was suppressed by ainsliadimer A (**1**) in a dose-dependent manner. We further investigated the effect of ainsliadimer A (**1**) on the processing of p100 to p52 triggered by the ectopic expression of NIK. Consistently, addition of ainsliadimer A (**1**) prevented NIK-mediated production of p52 (Supplementary Fig. 4). These results suggest that ainsliadimer A (**1**) also targets the non-canonical NF-κB pathway.

### Syntheses and biological evaluations of chemical probes

**Figure 3:**
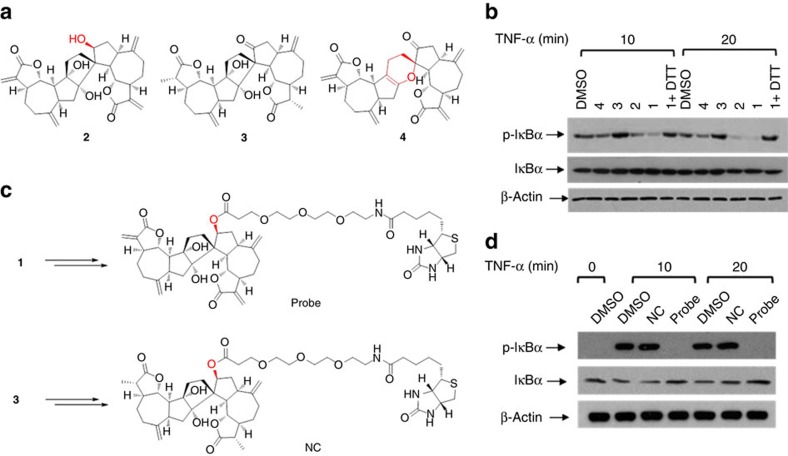
Syntheses and biological evaluation of chemical probes. (**a**) The chemical structures of analogues of ainsliadimer A (**1**). (**b**) The 293 T cells were preincubated with 8 μM of ainsliadimer A (**1**) or 8 μM of ainsliadimer A analogues for 1 h before stimulation with 10 ng ml^−1^ TNF-α. Cells were harvested at the indicated time points, and the total cell extracts were tested by western blot experiments for the occurrence of IκBα phosphorylation. (**c**) Chemical structures of biotin-labelled ainsliadimer A (positive probe, Probe) and biotin-labelled **3** (negative control, NC). (**d**) The 293 T cells were preincubated with 50 μM Probe or NC for 1.5 h before stimulation with 10 ng ml^−1^ TNF-α. Cells were harvested at the indicated time points, and the total cell extracts were tested by western blot experiments for the occurrence of IκBα phosphorylation. All experiments were repeated at least three times, and similar results were obtained each time.

To explore the functional target(s) of ainsliadimer A (**1**) associated with the NF-κB pathway, we prepared chemical probes for affinity purification. Initial structure and activity relationship studies enabled by total synthesis revealed that the hydroxyl analogue **2** fully retained the biological activity observed with ainsliadimer A (**1**), which suggested that a biotin tag could be attached to this hydroxyl group (Fig. 3a). In addition, the α, β-unsaturated enone moieties were shown to be essential for the biological activity as indicated by the totally inactive analogue **3** (Fig. 3b). This interesting result suggested that the enone moiety might be able to serve as a Michael acceptor to form a covalent bond with the thiol group of the cysteine residue. The inactive analogue **3** could conceivably be used to prepare a negative probe. On the basis of this information, we synthesized both a positive probe (Probe) and a negative probe (NC) from **2** and **3**, respectively, with biotin and a tri(ethyleneglycol) linker (Fig. 3c and Supplementary Information). The biological evaluations of Probe and NC showed that the biotin-tagged positive probe retained the ability to effectively block NF-κB activation at 50 μM, while the biotin-tagged negative probe completely lost the activity at the same concentration (Fig. 3d and Supplementary Fig. 5).

### Ainsliadimer A directly targets IKKα and IKKβ

**Figure 4:**
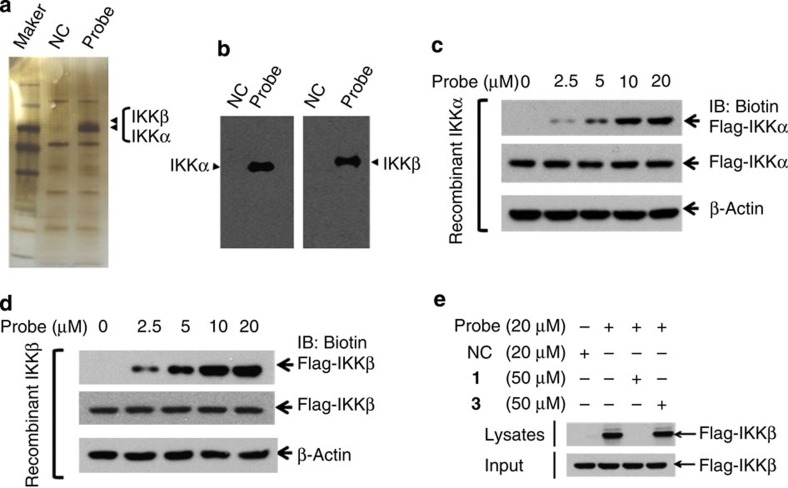
Ainsliadimer A (1) directly targets IKKα and IKKβ. (**a**,**b**) The 293T cell lysates were incubated with Probe or NC at 4 °C overnight, the lysates were used for streptavidin-agarose pull-down assays, and the precipitates were resolved by SDS–PAGE, followed by silver staining. The indicated bands were excised and analysed via mass spectrometry (**a**), or detected by western blotting for IKK proteins as indicated (**b**). (**c**,**d**) The recombinant IKKα and IKKβ proteins were incubated with Probe or NC for 1 h at 37 °C, followed by immunoblotting with biotin (upper band) or flag (lower band). (**e**) The recombinant IKKβ protein was incubated with Probe in the absence or presence of a twofold excess of unlabelled ainsliadimer A (**1**) or **3** for 1.5 h at 37 °C, and the mixtures were blotted for biotin or Flag. All experiments were repeated at least three times, and similar results were obtained each time.

To identify cellular proteins that interact with ainsliadimer A (**1**), we performed pull-down experiments using Probe and NC as positive and negative probes, respectively. The 293T-cell lysates were incubated with streptavidin agarose beads, which were pre-coupled with Probe or NC. The proteins precipitated by streptavidin agarose beads were resolved by sodium dodecyl sulfate–PAGE (SDS–PAGE) and stained with silver. As shown in (Fig. 4a), two clear bands observed at around 78 and 80 kDa were specifically precipitated by Probe, but not by NC. Peptide mass fingerprinting data analysis revealed that these two proteins were IKKα and IKKβ. We further used immunoblotting to monitor the presence of IKKα and IKKβ in the precipitates. Consistently, both IKKα and IKKβ were specifically pulled down by Probe, but not by NC (Fig. 4b). These data suggest that ainsliadimer A (**1**) targets IKKα and IKKβ or a complex that contains IKKα and IKKβ.

To determine whether ainsliadimer A (**1**) directly binds to IKKα and IKKβ, we generated recombinant IKKα and IKKβ proteins for further analysis. The 293T cells were transfected with Flag-IKKα or Flag-IKKβ plasmids, the recombinant Flag-IKKα or Flag-IKKβ was captured by anti-Flag agarose beads and then eluted off the beads using Flag peptide. The purified Flag-IKKα or Flag-IKKβ was incubated with DMSO or increasing concentrations of Probe, and then the mixtures were resolved by SDS–PAGE, followed by immunoblotting with streptavidin or anti-Flag antibodies. As shown in Fig. 4c, in the presence of Probe, a clear band with a molecular weight of around 80 kDa was detected by a streptavidin antibody. The signal intensity of this band increased with increasing concentrations of Probe, suggesting that there is a strong interaction between Probe and recombinant IKKα. Moreover, we also observed a similar interaction pattern between Probe and recombinant IKKβ (Fig. 4d). This binding could be out-competed by an excess of unlabelled ainsliadimer A (**1**), but not by **3** (Fig. 4e). Notably, ainsliadimer A (**1**) had no association with IKKγ/NEMO, another subunit of the IKK complex, and had no association with other components in the NF-κB pathway such as TAK1 or TAB1 (Supplementary Fig. 6). Taken together, these results demonstrate that ainsliadimer A (**1**) can efficiently and specifically interact with IKKα and IKKβ, and that the double bond is critical for its binding with IKKα and IKKβ.

### C46 of IKKα/β are critical for binding to ainsliadimer A

**Figure 5:**
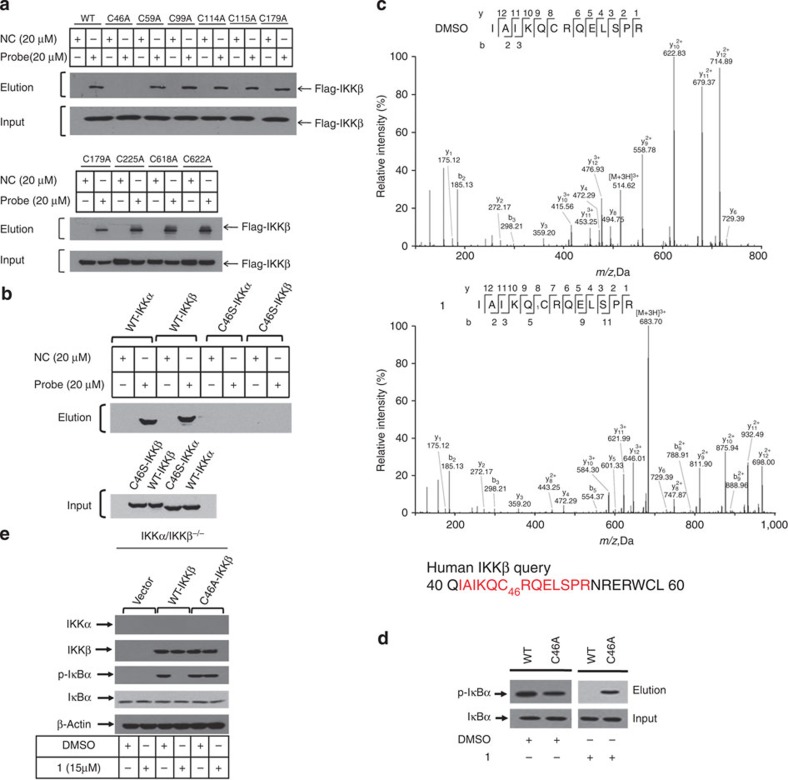
Cysteine 46 of IKKβ is critical for its binding to ainsliadimer A (1). (**a**) Recombinant WT-IKKβ and IKKβ-mutant proteins were incubated with Probe or NC at 37 °C for 1.5 h, followed by pull-down with streptavidin-agarose; the precipitates were then resolved by SDS–PAGE and blotted for biotin or flag. (**b**) Recombinant WT-IKKα, WT-IKKβ, C46S-IKKα or C46S-IKKβ was incubated with Probe or NC at 37 °C for 1.5 h, followed by pull-down with streptavidin-agarose; the precipitates were then resolved by SDS–PAGE and blotted for biotin or Flag. (**c**) MS/MS analysis of the recombinant IKKβ incubated with or without ainsliadimer A (**1**) for 3.5 h. (**d**) Effects of ainsliadimer A (**1**) on phosphorylation of IκBα *in vitro* using WT-IKKβ or C46A-IKKβ. Purified Flag-IκBα was used as the substrate and the indicated recombinant proteins were used as kinases. The mixtures were incubated with DMSO or ainsliadimer A (10 μM) for 1 h at 37 °C. Immunoblotting using the p-IκBα antibody reflects the kinase activity and the effects of ainsliadimer A (**1**). (**e**) IKKα/IKKβ^−/−^ double-knockout MEF cells were grown to 50% confluence, transfected with 200 ng of the indicated expression plasmids for 8 h, and then treated with ainsliadimer A (**1**) for 1.5 h. Shown are immunoblots of the total cell lysates using the indicated antibodies.

As ainsliadimer A (**1**) lost its inhibitory activity against NF-κB activation entirely when the enone moieties were saturated or in the presence of excess reducing agent dithiothreitol (DTT) (Fig.3b), the α,β-unsaturated moiety in ainsliadimer A (**1**) is most likely a reactive Michael acceptor, which forms a covalent bond with cysteine residues of its target proteins. Such a bond results in inactivation of the targeting enzymes, as previously demonstrated by natural products such as adenanthin^28^. Therefore, we speculated that the conserved cysteine residues in IKKα and IKKβ might be the binding sites of ainsliadimer A (**1**). We used BLAST analysis with the sequences of IKKα and IKKβ and found nine conserved cysteine residues (Supplementary Fig. 7). To evaluate which of these residues was critical for the binding of ainsliadimer A (**1**), we individually mutated these nine cysteine residues of IKKβ into alanine residues (Fig. 5a) using the primers detailed in Supplementary Table 1. Among these mutants, C46A was the sole mutant that completely lost the ability to interact with ainsliadimer A (**1**), while the other eight mutants retained high-affinity binding for ainsliadimer A (**1**) in a manner similar to wild-type (WT) IKKβ (Fig. 5a). These results indicated that C46 of IKKβ is essential for its binding to ainsliadimer A (**1**). As expected, mutation of C46 into serine in either IKKα or IKKβ abolished its corresponding interaction with ainsliadimer A (Fig. 5b).

To determine the specific residue modified by ainsliadimer A (**1**) in IKKβ, we incubated recombinant IKKβ protein with or without ainsliadimer A (**1**). After the reaction, the IKKβ protein was digested with trypsin and then analysed by LC-MS/MS. Database searching identified a peptide with calculated mass of 2,047.09 Da, which is 506.2 Da larger than the C46-containing peptide IAIKQCRQELSPR that has a calculated mass of 1,540.85 Da. The mass difference of 506.2 Da matches the molecular weight of a ainsliadimer A (**1**) molecule. Tandem mass spectrometry of this peptide revealed that a 506.2 Da mass shift occurred starting from the y8 to the y12 fragment ions (Fig. 5c), indicating that the C46 residue was covalently modified by ainsliadimer A (**1**). To further confirm this, two synthetic peptides derived from human IKKβ (#1 and #2) containing C46 were incubated with ainsliadimer A (**1**), and the resulting mixture was analysed by MALDI-TOF (Matrix-Assisted Laser Desorption/Ionization Time of Flight) mass spectrometry. As expected (Supplementary Fig. 8), these two synthetic peptides had a mass shift corresponding to a molecular weight of ainsliadimer A (**1**) or Probe after the reaction, indicating that ainsliadimer A (**1**) and Probe could modify C46 *in vitro*. When the C46 in peptide #1 and peptide #2 was replaced by alanine or serine (referred to as peptides 1A, 2A, 1S and 2S), however, the mass of these peptides remained the same after the reaction with either ainsliadimer A (**1**) or Probe (Supplementary Fig. 8). Contrastingly, the synthetic IKKβ C215 containing peptides #3 (AA sequence 202-220) and #4 (AA sequence 212-231) could not be modified by either ainsliadimer A (**1**) or Probe (Supplementary Fig. 8). Together with the mutation binding assays shown in Fig.5b, these results indicate that ainsliadimer A (**1**) binds to IKKβ by covalently modifying its C46 residue.

Ainsliadimer A (**1**) likely modifies IKKβ through Michael addition at C46. To verify the functional significance of this binding event, we performed *in vitro* kinase assays using WT-IKKβ or C46A-IKKβ recombinant proteins as the kinase, and purified Flag-IκBα protein as the substrate. As shown in Fig. 5d, both recombinant WT-IKKβ and C46A-IKKβ phosphorylated IκBα, and the signal intensity of IκBα phosphorylation was similar, suggesting that the replacement of C46 by alanine does not affect the kinase activity of IKKβ. In the presence of ainsliadimer A (**1**), WT-IKKβ-induced phosphorylation of IκBα was significantly suppressed, whereas ainsliadimer A (**1**) did not affect the phosphorylation of IκBα triggered by C46A-IKKβ. Moreover, expression of WT-IKKβ or C46A-IKKβ in IKKα/IKKβ double-knockout MEF cells restored the phosphorylation and degradation of IκBα, and addition of ainsliadimer A (**1**) blocked the phosphorylation and degradation of IκBα induced by WT-IKKβ, but not that induced by C46A-IKKβ (Fig. 5e). Collectively, these results indicate that ainsliadimer A (**1**) inhibits IKKβ by direct modification of its C46 residue. Meanwhile, *in vitro* kinase assays using purified recombinant IKKβ and IκBα showed 2 μM of ainsliadimer A (**1**) could totally inhibit IκBα phosphorylation as BMS-345541 did (Supplementary Fig. 9). We next investigated the ability of ainsliadimer A (**1**) to affect the association of IKKβ with IκBα. As shown in Supplementary Fig. 10, treatment of ainsliadimer A (**1**) reduced the association of IKKβ with IκBα. Thus, ainsliadimer A (**1**) is proven to be able to block the function of IKKβ in the NF-κB pathway.

### Ainsliadimer A is a novel allosteric inhibitor of IKKα/β

**Figure 6:**
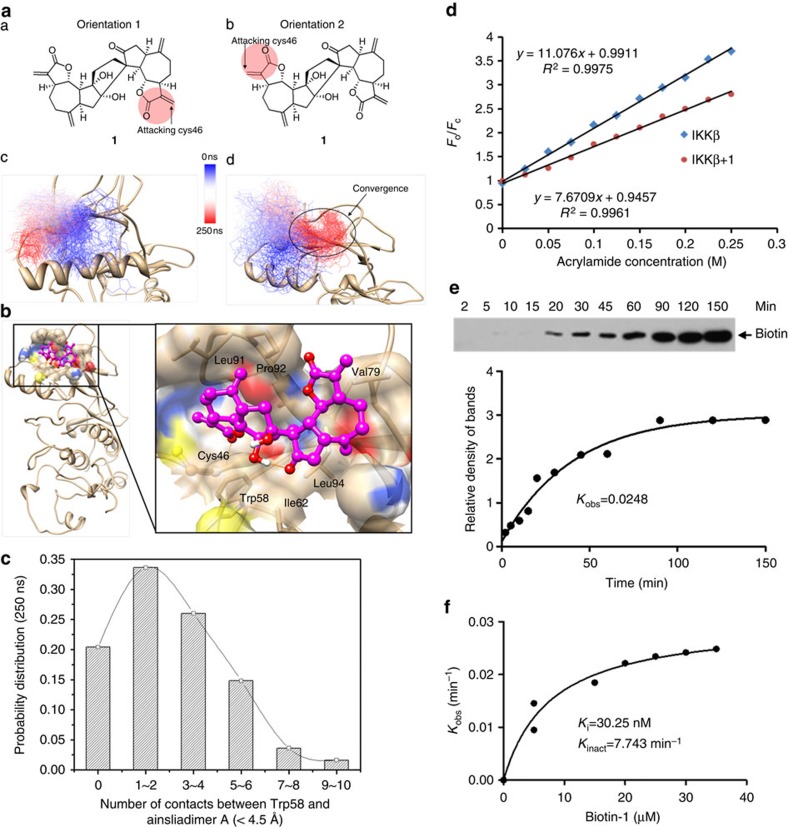
Ainsliadimer A (1) is a novel allosteric inhibitor of IKKβ. (**a**) (A and B) The two modelled structures used to predict that a Michael acceptor (highlighted using red circle) forms a covalent bond with C46; (C and D) Superimposed structures representing the 250 ns MD simulations in the Orientation-1 and the Orientation-2 systems, respectively. IKKβ is presented in a ribbon model in grey color (sulfur atom of C46 is shown as a yellow sphere), the ligand is shown in a thin line and coloured gradually from deep blue to deep red along the time series. Structural convergence was only observed in the Orientation-2 system, where the compound binds in a relatively stable conformation (highlighted in the black circle) after 180 ns and lasts for the remaining 70 ns (movie S1 records all of the MD simulation trajectories). (**b**) A representative view of a simulated binding complex. (A) The left side shows the global view of the entire IKKβ structure; the right side shows the focused view of ainsliadimer A (1) in the allosteric binding site. Ainsliadimer A (**1**) forms a hydrogen bond with the backbone of C46 and interacts favourably with several hydrophobic residues including Trp58, Ile62, Val79, Leu91, Pro92 and Leu94. (**c**) The number of close contacts (distance <4.5 Å) between any heavy atoms of ainsliadimer A (**1**) and Trp58. (**d**) Binding of ainsliadimer A (**1**) to IKKβ induced conformational change. Stern–Volmer plots for quenching of the intrinsic tryptophan fluorescence of IKKβ by acrylamide are shown. The detailed experimental protocol is described in the Online Methods. (**e**) The determination of *k*_obs_ for interaction of IKKβ (0.5 μM) with Probe (15 μM) for different times was calculated as described in Online Methods. (**f**) Plotting the *k*_obs_ value for binding of IKKβ as a function of ainsliadimer A (**1**) concentration.

To further explore how ainsliadimer A (**1**) inactivates IKKα/β, we conducted molecular dynamics (MD) simulations using the first crystal structure of IKKβ (ref. 29) to sample the conformational states of IKKβ on ainsliadimer A (**1**) binding. According to the computational study, the binding mode shows that a stable hydrogen bond forms between the backbone amide group of C46 and the Michael acceptor carbonyl group of ainsliadimer A (**1**) during the simulation; this may be involved in stabilizing the transition state of a Michael addition reaction (Fig. 6a). In addition, ainsliadimer A (**1**) forms favourable hydrophobic interactions with residues including Trp58, Ile62, Val79, Leu91, Pro92 and Leu94 (Fig. 6b), where an allosteric binding pocket was previously identified in 3-phosphoinositide-dependent kinase 1 (PDK1)^30^. In addition, based on our 250 ns MD simulation results, the binding of ainsliadimer A (**1**) introduces significant conformational changes to the ATP binding-site in IKKβ by displacing the conserved structural αC-helix (Supplementary Fig. 11a). As we observed in our simulation of ainsliadimer A (**1**)-bound IKKβ, such an allosteric regulation mechanism was also reported in the crystal structures of PDK-1 kinase, where a covalent inhibitor binds with Cys148 in the same allosteric binding pocket and induces a positional change in the C-helix by moving away from the ATP-binding site (Supplementary Fig. 11b)^30^,^31^.

Notably, ainsliadimer A (**1**) completely blocks solvent access to Trp58, which is located at the bottom of the allosteric pocket and interacts with ainsliadimer A (**1**) favourably throughout the simulation (Fig. 6c). Therefore, binding of ainsliadimer A (**1**) to the allosteric pocket can be experimentally assessed using the tryptophan fluorescence quenching technique, which has been widely used to demonstrate allosteric effects when co-crystal structures are not available^32^. Accordingly, we performed tryptophan fluorescence quenching studies to investigate whether the trypophan (W58) of IKKβ undergoes any changes on binding with ainsliadimer A (**1**). On the basis of the slope of the plot (Fig. 6d), we determined the Stern–Volmer constant for IKKβ quenching by acrylamide with or without the treatment of ainsliadimer A (**1**), which was 7.6709±0.2 and 11.076±0.2 M^−1^, respectively. This indicates that the binding of ainsliadimer A (**1**) to IKKβ partially protects W58 from the quencher by inducing conformational changes of the target protein. In other words, the process of ainsliadimer A (**1**) binding to IKKβ changes the disposition of W58 from being partially exposed to being in a more completely buried state. As expected, mutation of W58 into alanine in IKKβ abolished its corresponding interaction with ainsliadimer A (Supplementary Fig. 12). We propose that this conformational change induced by ainsliadimer A (**1**) might lead to the inhibition of IKKβ. In addition, it is worth noting that the downward curvature in the Stern–Volmer plot existed in the presence of ainsliadimer A (**1**); this was conceivably caused by the existence of both drug-bound and drug-free receptor populations, whose tryptophan was exposed to differing extents. Together, our data suggest that W58 on the α C-helix is required for the interaction between ainsliadimer A (**1**) and IKKβ. As displacement of α C-helix by a small molecule inhibitor or regulatory protein is able to affect ATP binding^33^, we further measured the ATP-binding kinetics of IKKβ in the absence or presence of ainsliadimer A (**1**). The result indicated that ainsliadimer A (**1**) showed ATP-competitive inhibitory effect on IKKβ (Supplementary Fig. 13). Moreover, ainsliadimer A (**1**) significantly suppressed WT-IKKβ-induced phosphorylation of IκBα. Collectively, these data indicate that C46 of IKKα/β likely acts as a novel allosteric regulation site of these enzymes.

Next, we investigated the binding kinetics of ainsliadimer A (**1**), as the conventional IC_50_ value could not well characterize the potency and selectivity for irreversible covalent inhibitors that we observed. We detected the extent of IKKβ binding at several time points for a number of Probe concentrations^28^. The progress of IKKβ binding with ainsliadimer A (**1**) at various concentrations showed time-dependent saturation (Fig. 6e), consistent with an irreversible mechanism of binding. The data were fit to determine the observed rate constants for binding (*k*_obs_) at each inhibitor concentration. Plotting these *k*_obs_ values as a function of Probe concentration revealed a saturation curve (Fig. 6f). These data support a two-step reaction mechanism for the inactivation of IKKβ by ainsliadimer A (**1**), involving the noncovalent reversible binding of IKKβ to ainsliadimer A (the initial binding step, *K*_i_), which places the moderately reactive electrophile of ainsliadimer A (**1**) close to a specific nucleophile on the protein, followed by the second chemical step that results in specific covalent linkage (*k*_inact_) leading to the inhibited complex^34^,^35^. On the basis of this model, the *k*_inact_ value for IKKβ binding with ainsliadimer A (**1**) was determined to be 7.743 min^−1^ (Fig. 6f). The *K*_i_ value for IKKβ binding to ainsliadimer A (**1**) was determined to be 30.25 nM (Fig. 6f). Overall, the binding process between ainsliadimer A (**1**) and IKKβ is effective (*k*_inact_/*K*_i_=4.26 × 10^6^ M^−1^ s^−1^), with good affinity (*K*_i_=30.25 nM) and specific reactivity (*k*_inact_=7.743 min^−1^) (Fig. 6e,f). We also systematically tested 340 human kinases through a contract service (http://www.reactionbiology.com/) to further confirm this specific binding to IKKβ. As shown in Supplementary Fig. 14, at the concentration of 200 nM, ainsliadimer A (**1**) did not show significant enzyme inhibition effects as compared with the inhibitory effect against IKKβ.

### Ainsliadimer A inhibits tumour growth

**Figure 7:**
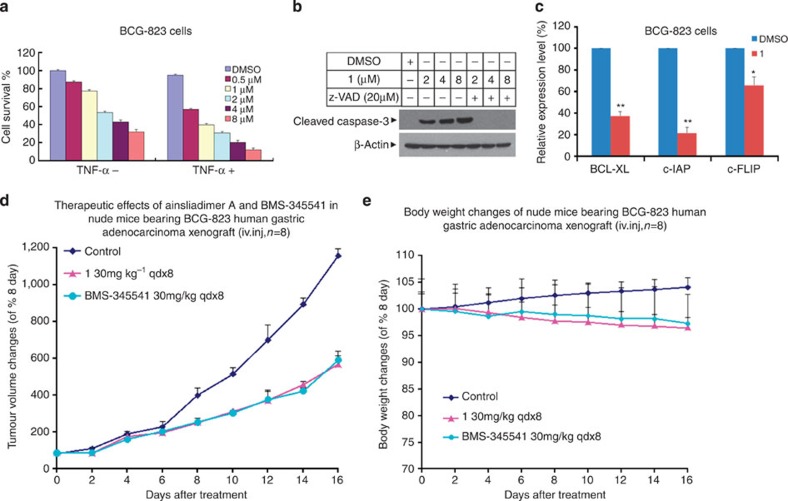
Ainsliadimer A (1) induces cell death of tumour cells and represses growth of xenograft tumours. (**a**) Ainsliadimer A (**1**) showed anticancer activity in BCG-823 cells. BCG-823 cells were seeded in a 96-well plate and then treated with TNF-α (10 ng ml^−1^) after pretreatment with the indicated concentration of ainsliadimer A (**1**) for 24 h. Cell viability was assayed by measuring ATP levels. (**b**) Ainsliadimer A (**1**) induced caspase-3 activation. BCG-823 cells were treated with or without the pan-caspase inhibitor z-VAD-FMK (40 μM) 1 h before the addition of 4 μM of ainsliadimer A (**1**), and then caspase-3 activation was measured by western blot using anti-cleaved caspase-3 antibodies. (**c**) Effects of ainsliadimer A (**1**) on NF-κB-regulated genes involved in apoptosis in BCG-823 cells. BCG-823 cells were treated with or without 4 μM ainsliadimer A (**1**) for 16 h, the expression of Bcl-XL, c-IAP and c-FLIP was quantified by real-time PCR. All the experiments were carried out in triplicate. The data are means±s.d. of the indicated experiments. *P<0.05 versus DMSO and **P<0.01 versus DMSO. (**d**,**e**) *In vivo* therapy with ainsliadimer A (**1**) in disseminated BCG-823 human gastric adenocarcinoma xenografts. Data plotted are the representative tumour size (**e**) or mouse body weight (**f**) of two rounds of experiments (*n*=8 for each group).

In addition to its crucial role in controlling immune responses, the NF-κB pathway has been shown to negatively regulate apoptosis and positively regulate the onset and progression of tumours^12^. Aberrant or constitutive NF-κB activation has been observed in many cancer cells, which usually exert increased resistance to chemotherapy. Inhibition of NF-κB results in an increased chemosensitivity of cancer cells to therapeutic drugs as well as cell death. To assess the anticancer effects of ainsliadimer A (**1**), we treated gastric adenocarcinoma BCG-823 cells with ainsliadimer A (**1**) alone, TNF-α alone or with a combination of ainsliadimer A (**1**) and TNF-α for a period of 24 h. As shown in Fig. 7a, pretreatment with TNF-α resulted in a synergistic repression of cell viability. We further examined whether ainsliadimer A (**1**) triggers apoptosis by measuring caspase-3, which is activated via proteolytic cleavage during apoptosis. In BCG-823 cells treated with ainsliadimer A (**1**), we detected cleaved caspase-3 that could be blocked by z-VAD, a pan-caspase inhibitor (Fig. 7b). Furthermore, we found that ainsliadimer A (**1**) suppressed the activation of NF-κB (Supplementary Fig. 3) and downregulated anti-apoptotic genes including c-FLIP, c-IAP and BCL-XL (Fig. 7c). These results demonstrate that the effects of ainsliadimer A (**1**) on the NF-κB pathway facilitate apoptosis of cancer cells. Finally, we evaluated the *in vivo* effects of ainsliadimer A (**1**) in tumour growth. Nude mice bearing a BCG-823 human gastric adenocarcinoma xenograft were treated with Vehicle or ainsliadimer A (**1**) (30 mg kg^−1^, qdx8) or BMS-345541, a widely used IKKβ inhibitor (30 mg/kg, qdx8). As shown in Fig. 7d, significant xenograft tumour remission was observed after 16 days in all (eight of eight) of the mice treated with ainsliadimer A (**1**), as compared with the Vehicle-treated mice. Notably, ainsliadimer A (**1**) exerted a comparable anti-tumour effect to that of the therapeutic drug BMS-345541. Importantly, the mice did not display any discernible side effects or significant changes in body weight during administration of ainsliadimer A (Fig. 7e). Collectively, these results demonstrate that the inhibition of NF-κB by ainsliadimer A (**1**) resulted in significant death of human cancer cells and suppression of tumour growth with minimal undesired toxicities, thus implicating ainsliadimer A (**1**) as a novel IKK inhibitor with promising potential for anticancer and anti-inflammatory therapeutics.

## Discussion

NF-κB transcription factors play a critical function in the regulation of immune responses, inflammation, cell proliferation and survival. Aberrant activity of NF-κB is associated with tumour progression as well as auto-immune and inflammatory diseases. Therefore, crucial regulators in the NF-κB pathway such as IKKα/β are considered as promising therapeutic targets. We show here that a natural product ainsliadimer A (**1**) specifically binds to IKKα/β and subsequently blocks both canonical and non-canonical NF-κB activation.

Ainsliadimer A (**1**) was originally isolated from a Chinese herb^22^. Although ainsliadimer A (**1**) has been shown to suppress LPS-mediated NO production^22^, its precise molecular targets and mode of action remained uncharacterized. LPS is known to activate TLR4 and to result in NF-κB activation and type I IFN responses. Interestingly, we found that ainsliadimer A (**1**) specifically blocked LPS-induced NF-κB activation, but not type I IFN responses. Moreover, ainsliadimer A (**1**) exerts potent inhibition of NF-κB activation triggered by different stimuli such as TLR3 ligand poly(I:C) and TNF. Using biotin-labelled 1 as a probe, we found that ainsliadimer A (**1**) directly targeted IKKα and IKKβ, but not other components of the NF-κB pathway such as IKKγ/NEMO, TAK1 and TAB1, suggesting that an exclusive binding mechanism is shared by IKKα and IKKβ.

Ainsliadimer A (**1**) is a structurally unique and complex sesquiterpene lactone dimer with α, β-unsaturated enone moieties that may serve as Michael acceptors. Consistently, our mutagenesis and mass spectrometry results demonstrated that ainsliadimer A (**1**) formed a covalent bond with the conserved C46 of IKKα/β. Through both computational and experimental studies we further uncovered, remarkably, a novel putative allosteric binding pocket around C46 of IKKβ that could explain the high-binding specificity of ainsliadimer A (**1**) bound to IKKβ. Notably, the tryptophan fluorescence quenching results provided direct and strong evidence to support this novel allosteric effect. In addition, ainsliadimer A (**1**) was shown to be an irreversible inhibitor that exhibited specific and potent kinase inhibition activity against IKKβ with *k*_inact_ /*K*_i_ of 4.26 × 10^6^ M^−1^ s^−1^.

Many small molecule inhibitors that interfere with IKKs have been previously described^12^,^21^, but the precise modifications of the target by most of these inhibitors remain obscure. To our knowledge, ainsliadimer A (**1**) represents the first small molecule that blocks NF-κB by directly targeting C46 of IKKα/β. The putative allosteric site around C46, most likely unique to IKKα/β, confers the high selectivity of ainsliadimer A (**1**) for IKKα/β, thereby tending to have low non-specific toxicity and a high therapeutic index. Further, in both *in vitro* and *in vivo* studies, ainsliadimer A (**1**) was shown to be a promising agent against the survival of cancer cells, tumour growth and endotoxin-mediated inflammatory responses. It is tempting to speculate that this unique inhibitory effect of ainsliadimer A (**1**) on IKKα/β should be further exploited for the elucidation of uncharacterized mechanisms of IKKα/β activation, as well as the development of new anticancer and anti-inflammatory therapies.

## Methods

### Chemical synthesis

The syntheses of **2**, **3**, the positive probe (Probe) and the negative probe (NC) were carried out according to Supplementary Fig 16 and the Supplementary Methods. NMR spectra of the compounds involved in this work were shown in Supplementary Figs 17–28. Ainsliadimer A (**1**) and **4** were synthesized as described in ref. 23.

### Reagents

DMEM medium, phosphate buffered saline (PBS), trypsin and penicillin/streptomycin were purchased from GIBCO. ATP, MgCl_2_, fetal bovine serum (FBS), BMS-345541, DTT, DMSO and LPS were purchased from Sigma. The Cell Titer-Glo Luminescent Cell Viability Assay kit was purchased from Promega. The Protein Assay kit was purchased from Bio-Rad. The HTRF KinEASE kit was purchased from Cisbio. IKKβ kinase protein was purchased from Carna Biosciences. RANKL was purchased from Sino Biological. Human IκBα full length protein was purchased from Abcam (ab59981).

### Plasmids

The PCS2-3Flag-IKKα, PCS2-3Flag-IKKβ, PCS2-3Flag-TAK1, PCS2-3Flag-NEMO, PCS2-3Flag-TAB1, PCS2-3Flag-NIK, myc-p100, Flag-IκBα plasmids were kindly provided by Dr Feng Shao and his coworkers (NIBS).

### Antibodies

The following antibodies were used for western blotting: p-IκBα (1:1,000 dilution, Cell Signaling, 9246), IκBα (1:1,000 dilution, Cell Signaling, 9242), IKKα (1:1,000 dilution, Cell Signaling, 2682), IKKβ (1:1,000 dilution, Cell Signaling, 2684), p-IKKα/β (1:1,000 dilution, Cell Signaling, 2697), p100 (1:1,000 dilution, Cell Signaling, 4882), H1 (1:1,000 dilution, Santa Cruz Biotechnology, sc-8030), Biotin (1:10,000 dilution, Sigma, A4541), Flag (1:10,000 dilution, Sigma, A8592), myc (1:3,000 dilution, Sigma, C3956), p-IRF3 (1:1,000 dilution, Cell Signaling, 4947) and β-actin (1:3,000 dilution, Cell Signaling, 4967).

### Cell cultures

Human cervical cancer HeLa, human glioblastma T98G, human gastric adenocarcinoma BCG-823, human embryonic kidney 293 T and mouse macrophage Raw264.7 cells were obtained from ATCC. These were cultured in DMEM medium supplemented with 10% (v/v) heat-inactivated FBS in a humidified incubator at 37 °C and 5% CO_2_/95% air (v/v). All media were supplemented with 10% FBS (Invitrogen) and 100 units ml^−1^ penicillin/streptomycin (Hyclone).

### Cell survival assay

The cell survival assay was performed using the Cell Titer-Glo Luminescent Cell Viability Assay kit (Promega) according to the manufacturer's instructions. Luminescence was recorded with a TecanGENios Pro plate reader.

### NF-κB activation

Cells were pre-incubated with various concentrations of compound ainsliadimer A (**1**) for 1 h before stimulation with the indicated concentrations of TNF-α, 20 μg ml^−1^ Poly(I:C) or 200 ng ml^−1^ LPS and then harvested at the indicated time points. Total cell extracts were tested by western blotting for the occurrence of IκBα phosphorylation. DMSO and BMS-345541 were chosen as negative and positive controls, respectively.

### NF-κB/p65 nuclear translocation immunofluorescence

HeLa Cells were grown to 50–70% confluence in a glass chamber and pretreated with DMSO or 8 μM ainsliadimer A (**1**) for 1.5 h and then stimulated by 20 ng ml^−1^ TNF-α for 30 min. Cell treatment was terminated by washing with PBS, followed by fixation in freshly prepared 4% paraformaldehyde in PBS for 10 min. The fixed cells were washed three times with PBS and then permeabilized in 0.25% Triton X-100 in PBS for 10 min. After blocking with 2 mg ml^−1^ BSA for 1 h at room temperature, we added a P65 antibody at 1:1,000 dilution at 4 °C overnight, washed three times with PBS, then added 1:500 dilution of secondary AlexaFluor488 antibody for 1 h at room temperature in the dark. Finally DAPI was used to stain the nuclei at 37 °C for 30 min in dark. Microscopy was performed using a Nikon eclipse 80i.

### Western blot analysis

Cell pellets were collected and resuspended in lysis buffer (20 mM Tris-HCl, pH 7.4,150 mM NaCl, 10% glycerol, 1% Triton X-100, 1 mM Na_3_VO_4_, 25 mM β-glycerol-phosphate, 0.1 mM PMSF, with a complete Roche protease inhibitor set and a Sigma phosphatase inhibitor set). The resuspended cell pellet or tissue was vortexed for 10 s, incubated on ice for 20 min, and centrifuged at 20,000 × *g* for 20 min. The supernatants were collected for western blot analysis or immunoprecipitation. Images have been cropped for presentation. Full-size images are presented in Supplementary Fig. 15.

### Pull-down and MS analysis of 1-bound proteins

The 293 T cells were plated on 10-cm Petri dishes and grown to confluence for 1 day. The cells were harvested and lysed in lysis buffer as described above. Probe or NC was preincubated with streptavidin agarose (Invitrogen) overnight at 4 °C. The beads were then washed three times in lysis buffer. The ainsliadimer A-bound agarose was then incubated with cell lysates overnight at 4 °C. The following day, the beads were washed six times with lysis buffer, then the immunoprecipitations were eluted off the beads using low pH elution buffer at 4 °C for 15 min. Acid elution was neutralized by adding 1/20 volume of 1 M Tris-HCl, pH 9.4, and the preparations were finally boiled in SDS loading buffer. The bead-bound proteins were separated by SDS–PAGE and visualized by silver staining. The protein-containing band in the gel was excised, followed by in-gel digestion and analysis by LC-MS/MS.

### Preparation of the wild-type and the site-mutated IKKα or IKKβ

Human IKKα or IKKβ were cloned into a PCS2 vector containing a 3 × Flag tag sequence at the N-terminal region. Site-directed mutagenesis was performed with the QuikChange site-directed mutagenesis kit (Stratagene) using Flag tag-IKKα or Flag tag-IKKβ as a template. These proteins were expressed in 293 T cells and subsequently purified.

### MALDI-TOF analysis

The reaction mixtures of peptides or proteins were analysed by matrix-assisted laser desorption/ionization-time of flight mass spectrometry with an Autoflex II MALDI-TOF/TOF mass spectrometer equipped with a nitrogen pulsed laser (Bruker). In brief the peptide mixtures were loaded on C18 Zip Tips separately, washed with 0.1%TFA, eluted with 2, 5-dihydroxybenzoic acid solution (Agilent), then spotted on a Bruker MTP 384 massive stainless steel MALDI target. The matrix spots were allowed to dry at room temperature. Peptide Mass Spectra were acquired in positive reflectron mode with pulsed ion extraction.

### Molecular modelling

The crystal structure of IKKβ (PDB entry: 3QA830) was used to generate initial binding complexes. The MD simulation was performed with the Gromacs 4.0.7 package^35^,^36^. During the simulation, we used the Amber99SB protein force field^37^ for proteins and general amber force field^38^,^39^ for small molecules. The complex was placed in a PBC box with at least 12 Å between solvent and solutes. The whole system was neutralized with both sodium and chlorine ions. Distance-dependent harmonic restraint was placed onto the sulfur atom of C46 and the Michael acceptor carbon atom of the ainsliadimer A with the initial distance value set to be 10 Å.

### Tryptophan fluorescence quenching studies

Twenty-five micromolars of ainsliadimer A (**1**) or DMSO was preincubated with 5 μM IKKβ for 90 min and titrated with 2.5 μl of 1 M acrylamide; we monitored the tryptophan florescence signal intensity by using 340 nm emission and 295 nm excitation. At the same time, the background signal was corrected by using a paired control that lacked protein. After using the Stern–Volmer equation to analyse *F*_0_/*F*, we compared the slopes from IKKβ with or without the treatment of ainsliadimer A (**1**). For the Stern–Volmer equation:


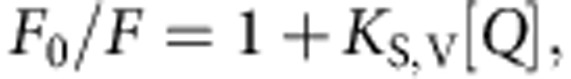


where *F*_0_ is the emission intensity of the protein without adding quencher, *F* is the emission intensity of the protein at a given quencher concentration, *K*_S,V_ is the Stern–Volmer constant for quenching, given by the slope when data are plotted as *F*_0_ /*F* versus [*Q*].

### The ATP binding kinetics of IKKβ

We used an HTRF KinEASE kit (Cisbio Bioassays, Bagnols/Ceze Cedex, France) to measure the ATP binding kinetics of IKKβ. After optimizing the conditions of IKKβ according to the kit protocol, the assays were carried out in the low volume, black 384-well plates (3676 from Corning life Science, MA), with a 10 μl assay volume containing varied concentration of ATP, non-limiting STK Substrate 2-biotin and 1 ng μl^−1^ of IKKβ. Following incubation at 37° for 1.5 h, the reaction was stopped with buffer EDTA, which contained the detection reagents, strepavidin-XL 665 and STK-antibody labelled with Eu3+-cryptate. The resulting TR-FRET signal, calculated as the fluorescence ratio at 665/620 nm, was read on the Envision. The *K*_m_ for ATP was determined with the Michaelis–Menten equation fits in GraphPad Prism 5.0.

### Kinetic determination of the IKKβ-Ainsliadimer A (1) interaction

The specific interaction between IKKβ and Probe followed two steps: in the initial binding step, ainsliadimer A (**1**) moves close to specific nucleophile residues of IKKβ to form the initial encounter complex IKKβ: ainsliadimer A (**1**), and *K*_i_ is the apparent dissociation constant for the initial IKKβ: ainsliadimer A (**1**) complex; in the second chemical step, the formation of specific covalent linkage (*k*_inact_) between ainsliadimer A (**1**) and IKKβ resulted in the inhibited complex and *k*_inact_ the rate constant for IKKβ- ainsliadimer A (**1**) formation at saturating ainsliadimer A (**1**). To get the *K*_i_ and *K*_inact_ value, we incubated IKKβ with a large excess of ainsliadimer A (**1**) for different time periods and then analysed the streptavidin-HRP signal intensity quantified by densitometry. During this process, we performed scanning at the optimal exposure time to ensure that band intensity was proportional to the concentration of protein present. Under the pseudo-first order experimental conditions, the reaction proceeds as in these equations:


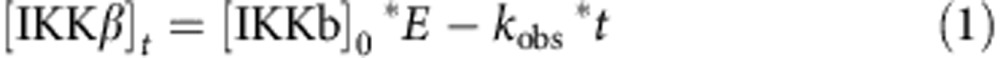



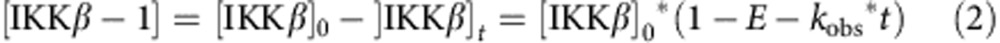


In equation (1), [IKKβ]_0_ is the total concentration of IKKβ added to the solution and [IKKβ]_t_ is the concentration of IKKβ at time *t*.

We initially obtained the reaction rate constants of *k*_obs_ at different ainsliadimer A (**1**) concentrations by fitting to equation (2). We then obtained the value of *k*_inact_ and *K*_i_ by fitting *k*_obs_ values to equation (3)


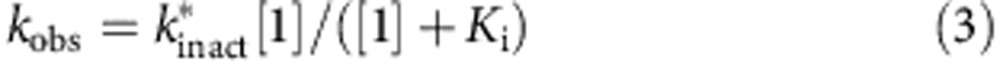


### RNA extraction and real-time PCR analysis

Cells were lysed and total RNA was extracted using the TRIzol Reagent (Invitrogen). Synthesis of cDNA was done with 1 μg of RNA from oligo (dT)-20 primers using the Superscript II first strand synthesis system (Invitrogen). Real-time PCR was performed using specific primers and the SYBR Green ROX Mix (Thermo Scientific). The sequence of the primers is given in the Supplementary Table 2. All experiments were performed in triplicate, and data were normalized to the housekeeping gene *GAPDH*.

### Analysis of levels of LPS-induced pro-inflammatory cytokines

Cytokines were assessed by measuring serum concentrations. C57 Male mice at 7–8 weeks of age were randomly assigned into five groups: the PBS, LPS, LPS+Vehicle, LPS+ ainsliadimer A (**1**) and LPS+BMS-345541 groups. The mice were administered intravenously with ainsliadimer A (**1**) (25 mg kg^−1^) (LPS+ ainsliadimer A group), BMS-345541(25 mg kg^−1^) (LPS+BMS-345541group), Vehicle (LPS+Vehicle group) or PBS(PBS group) for 90 min, and then the mice (LPS group, LPS+1,LPS+Vehicle group) were given intraperitoneal injection of LPS (1.6 mg kg^−1^). Mice in those groups were killed and their serum was separated from clotted blood at 3.5 h following administration of LPS. Serum was stored at –80 °C, and concentrations of cytokine IL-1β and IL-6 were measured by sandwich ELISA using commercially available reagents according to the manufacturer’s instructions (Beijing Gersion Bio-Technology). The concentration of cytokine TNF-α was measured using a Mouse Inflammatory Cytometric Bead Array (BD Biosciences, Palo Alto, CA) according to the manufacturer’s protocol. For each group, plasma samples were obtained from at least six mice and analysed in duplicate.

Meantime, we also got the spleen extracts from each mice and then analysed the protein levels of p-IκBα, IκBα and β-Actin by western blot.

### Establishment of subcutaneous and disseminated xenografts

Female nude (nu/nu) mice (6–7 weeks old) were purchased from Vital River Company and acclimated for 1 week before the start of the experiment. All animal experiments were performed in accordance with protocols by the Institutional Animal Care and Use Committee at National Institute of Biological Sciences (NIBS). Exponentially growing BCG-823 cells were suspended in PBS and mixed in a 1:1 ratio with Matrigel 0.1 ml suspension containing 2.5–5 × 10^6^ cells, and was injected subcutaneously on both the left and the right flank of each mouse above the hind limb. After tumour volumes reached 100 mm^3^, the mice were divided into experimental cohorts of eight mice each. The mice were received the intravenous injection of vehicle, ainsliadimer A (30 mg kg^−1^, qdx8) or BMS-345541, a widely used IKKβ inhibitor (30 mg kg^−1^, qdx8). Control mice were treated with the same amount of vehicle under the same protocol. Tumour volume was calculated by using the formula mm^3^=4/3*πr*^3^, where *r*=(length+width)/4, measuring the two largest perpendicular axes of the tumour.

### Statistical analysis

All data are expressed as the mean ±s.e.m. Results are representative examples of three individual experiments. Inferential statistical analyses were performed with a Student’s *t*-test (*P<0.05; **P<0.01; ***P<0.001; NS, not significant). Graph Pad Prism was used for analysis.

## Author contributions

X.L. and S.H. designed the study; T.D. and X.W. performed and analysed the biological experiments; C.L. and L.D. performed chemical synthesis; X.Z. performed and analysed the xenograft studies; R.C. performed the docking experiments under the guidance of N.H.; L.L. and S.C. performed MS experiments; X.L., S.H., T.D. and C.L. wrote the manuscript. X.L. and S.H. guided all of the aspects of this study.

## Additional information

**How to cite this article**: Dong, T. *et al*. Ainsliadimer A selectively inhibits IKKα/β by covalently binding a conserved cysteine. *Nat. Commun.* 6:6522 doi: 10.1038/ncomms7522 (2015).

## Supplementary Material

Supplementary Information Supplementary Figures 1-28, Supplementary Tables 1-2, Supplementary Notes 1-2, Supplementary Methods and Supplementary References.
