# Supplementary material for: Ainsliadimer A selectively inhibits IKKα/β by covalently binding a conserved cysteine
Source: Nat Commun. 2015 Mar 27;6:6522. doi: 10.1038/ncomms7522 (PMC4389228; doi:10.1038/ncomms7522)
Supplement: Supplementary Information — Supplementary Figures 1-28, Supplementary Tables 1-2, Supplementary Notes 1-2, Supplementary Methods and Supplementary References. [file ncomms7522-s1.pdf]

Supplementary Figures

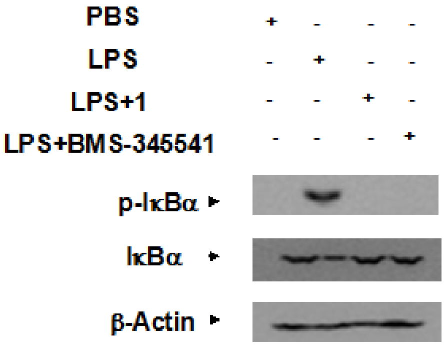

Supplementary Figure 1. Ainsliadimer A (1) inhibited IκBα phosphorylation in spleen extracts. The mice were received intravenous injection of DMSO, **1** (25 mg/kg) or BMS-345541 (25 mg/kg) following LPS challenge. Then extracts the spleen tissue and analyzed the protein levels of p-IκBα, IκBα, and β-Actin by western blot.

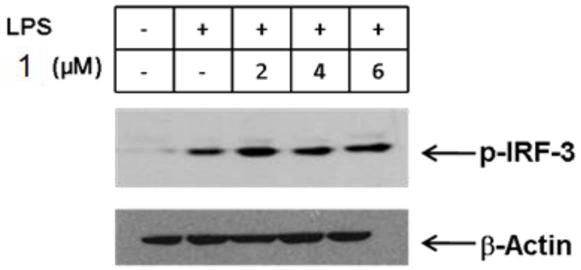

Supplementary Figure 2. The effect of ainsliadimer A (1) on LPS induced IRF3 phosphorylation. Raw 264.7 cells were pre-incubated with various concentrations of **1** for 1 h prior to stimulation with 20 ng/mL LPS, then cells were harvested after 60 minutes and total cell lysates were tested by western blot experiments for the occurrence of IRF3 phosphorylation. All experiments were repeated at least three times with the similar results.

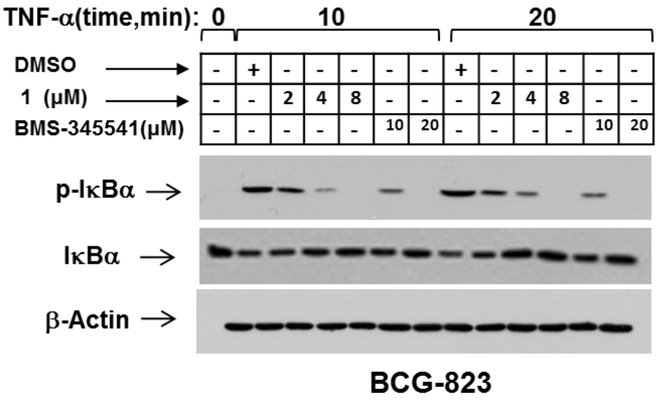

Supplementary Figure 3. Ainsliadimer A (1) blocks NF-κB activation induced by TNF-α. BCG-823 cells were pre-incubated with the indicated concentration of **1** for 1 hour prior to stimulation with 10 ng/mL TNF-α. Cells were harvested at the indicated time points and total cell extracts were tested by western blot experiments for the occurrence of IκBα phosphorylation. All experiments were repeated at least three times with the similar results.

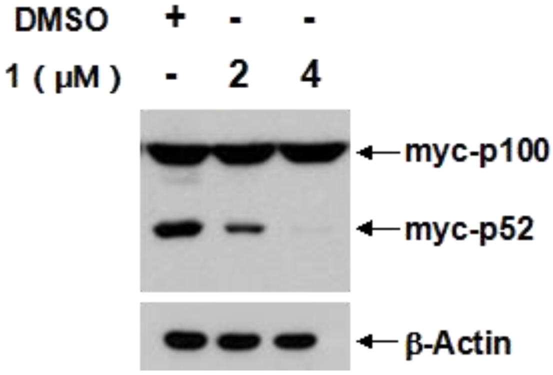

Supplementary Figure 4. The effect of ainsliadimer A (1) on the phosphorylation of p100 in the non-canonical NF-κB pathway processing into p52 induced by NIK. 293T cells were co-transfected with 100 ng NIK expression plasmid together with 2 μg myc-p100 expression plasmid. At 24 h post-transfection, cells were treated with DMSO or 4 μM **1** for 2 hours. Cells were harvested and total cell extracts were subjected to immunoblotting with myc or β-Actin. All experiments were repeated at least three times with the similar results.

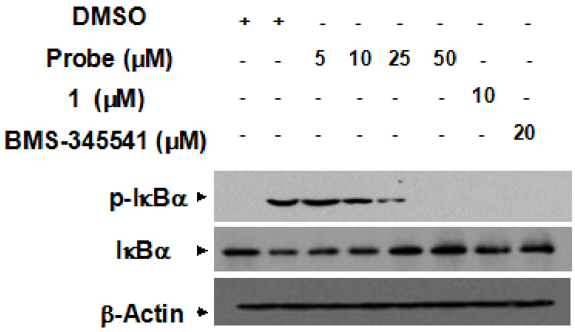

Supplementary Figure 5. Probe blocks NF-κB activation induced by TNF-α. 293T cells were pre-incubated with different concentration of Probe for 1.5 h prior to stimulation with 10 ng/mL TNFα. Cells were harvested and the total cell extracts were tested by western blot experiments for the occurrence of IκBα phosphorylation. All experiments were repeated at least three times, and similar results were obtained each time.

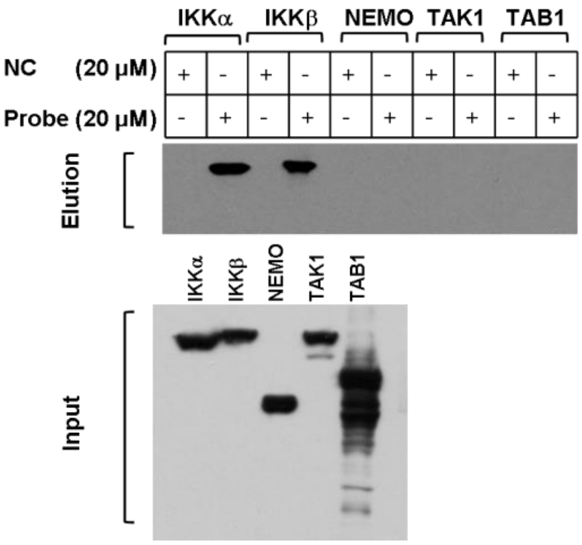

Supplementary Figure 6. Ainsliadimer A (1) specifically binds with IKKα and IKKβ in NF-κB signaling pathway. 293T cells were transfected with Flag-tagged IKKα, IKKβ, NEMO, TAK1 or TAB1. After 24 hours, the equally amounts of cell lysates were incubated with NC or Probe at 4°C overnight, followed by pull-down with streptavidin-agarose. The precipitates were resolved by SDS-PAGE and blotted for Flag. All experiments were repeated at least three times with similar results.

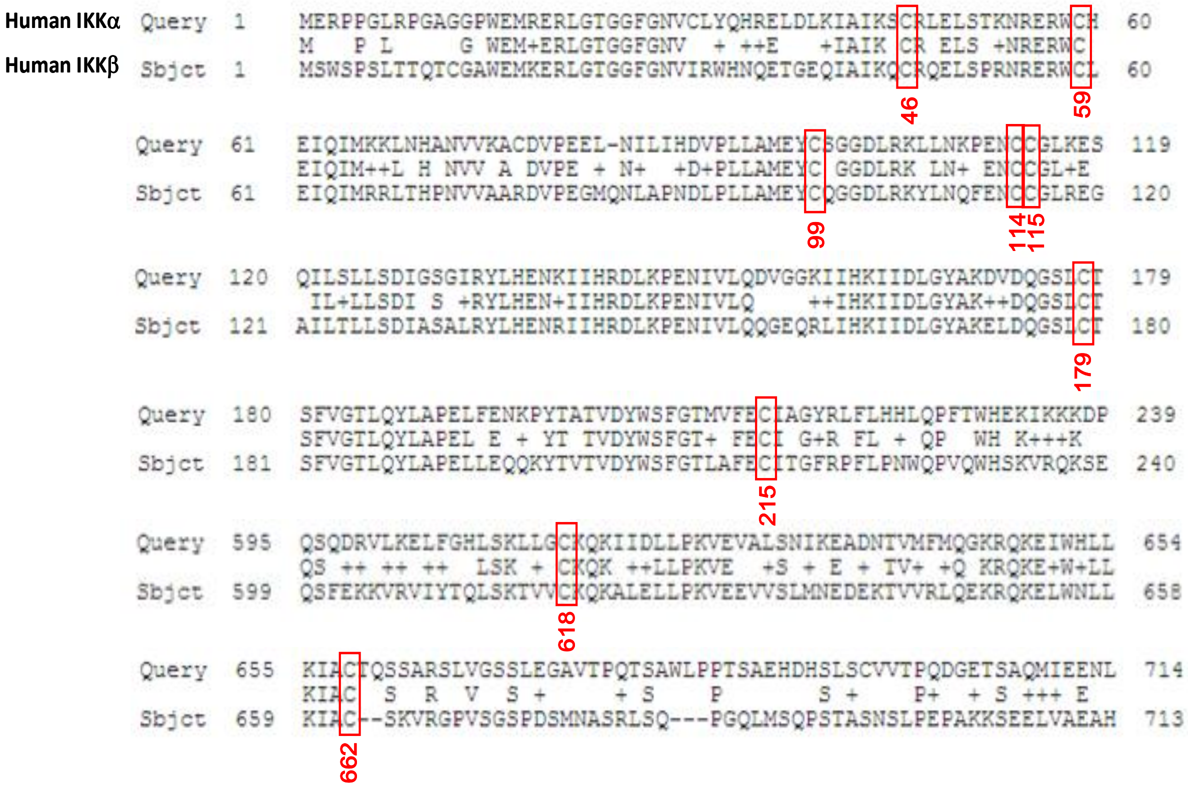

Supplementary Figure 7. Conserved cysteine residues in IKKα and IKKβ . Alignment of the amino acid sequence of human IKKα and IKKβ. Red box indicates conserved cysteine residues in human IKKα and IKKβ.

Peptide name	Peptide sequence	Binding with 1	Binding with Probe
**#1**	**IAIKQCRQELSPR**	**YES**	**YES**
**1A**	**IAIKQARQELSPR**	**NO**	**NO**
**1S**	**IAIKQSRQELSPR**	**NO**	**NO**
**#2**	**WHNQETGEQIAIKQCR**	**YES**	**YES**
**2A**	**WHNQETGEQIAIKQAR**	**NO**	**NO**
**2S**	**WHNQETGEQIAIKQSR**	**NO**	**NO**
**#3**	**TVDYWSFJTLAFECITGFR**	**NO**	**NO**
**#4**	**HFACGQEDTYSNMLKIWVRP**	**NO**	**NO**

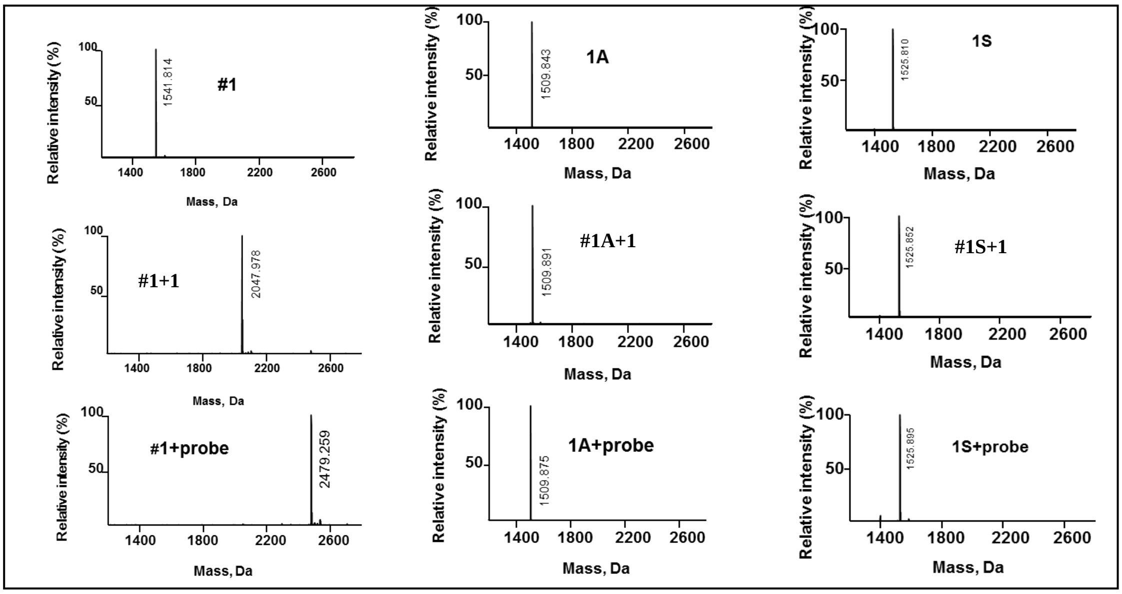

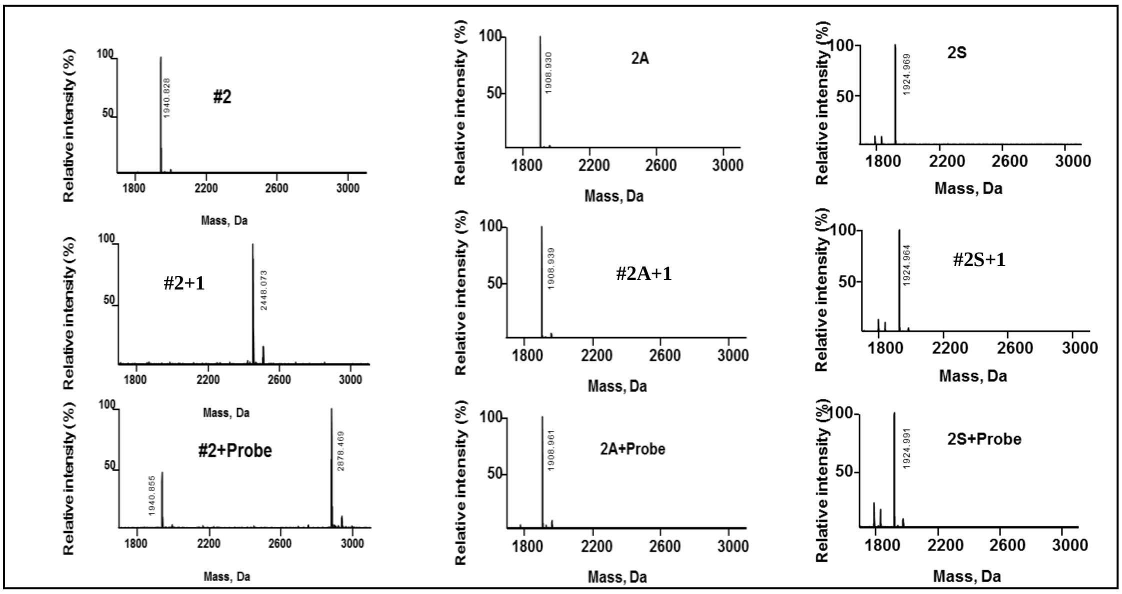

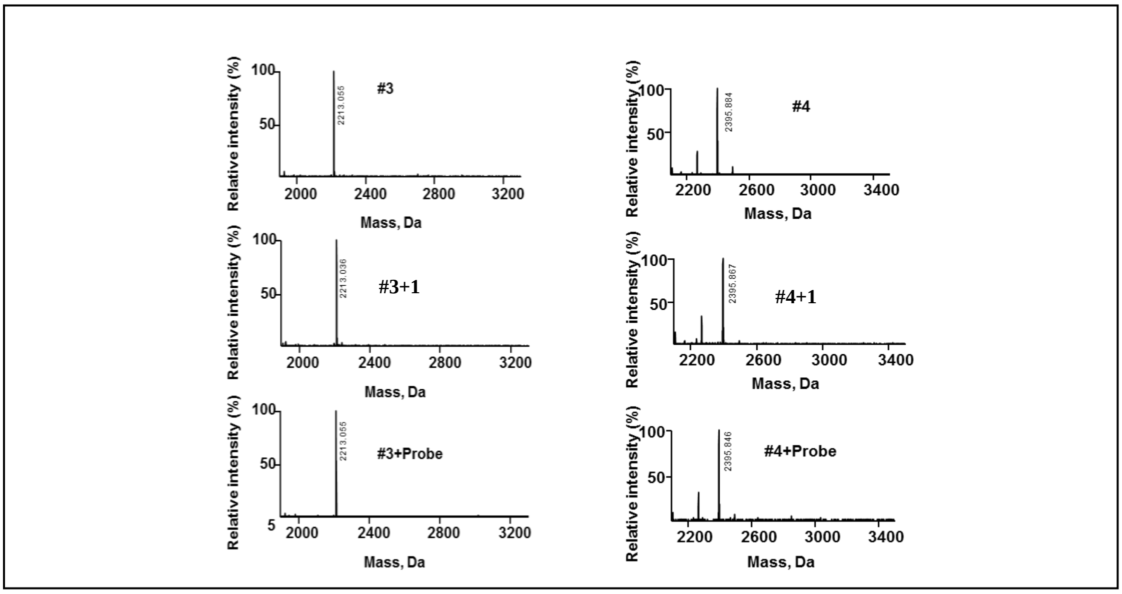

Supplementary Figure 8. The associations of ainsliadimer A **(1)** with or without the indicated synthetic peptides was determined by MALDI-TOF (Matrix-Assisted Light Desorption/Ionization Time of Flight) analysis. 1 mM synthetic peptides were incubated with DMSO, **1** (1 mM) or Probe (1 mM) for 6 h at 37°C, and then the peptide mixtures were analyzed by MALDI-TOF. The table lists six synthetic peptides including the sequence information and their ability to bind with **1** or Probe.

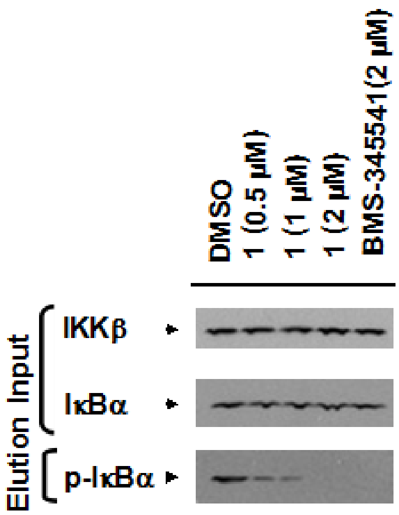

Supplementary Figure 9. Effects of ainsliadimer A (1) on phosphorylation of IκBα in vitro using bacterially expressed IKKβ and IκBα. Recombinat IkBa proteins were used as the substrate and recombinant IKKβ proteins were used as kinases. The mixtures were incubated with DMSO, **1** or BMS-345541 for 1h at 37°C. Immunoblotting using the p-IκBα antibody reflects the kinase activity and the effects of **1**.

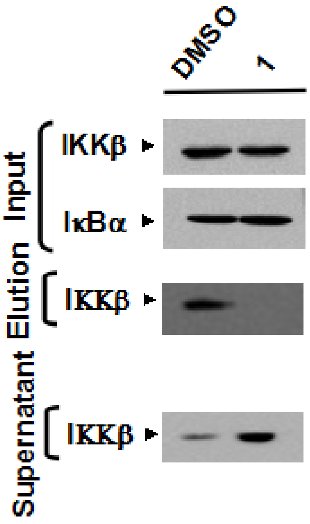

Supplementary Figure 10. Effects of ainsliadimer A (1) on IKKβ-IκBα interaction in vitro. GST-fused IκBα recombinat protein precoupled with glutathione-Sepharose 4B was incubated with IKKβ proteins at room temperature for 1 h with or without **1** (4 μM). After centrifugation, the supernatants were removed. The beads were then washed twice, eluted and subjected to SDS–PAGE analysis with anti-IKKβ and anti-IκBα antibodies. All pull-down experiments were repeated three times with consistency.

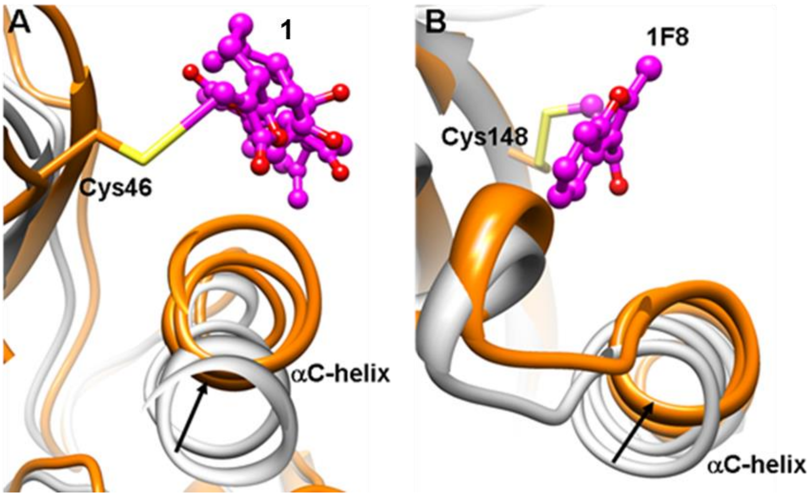

Supplementary Figure 11. (a) Superimpose of simulated structures of Ikkβ at 0 ns (grey) and 250 ns (orange); (b) superimpose of crystal structures of PDK1 at apo state (grey) and in complex with an allosteric inhibitor 1F8 (orange).

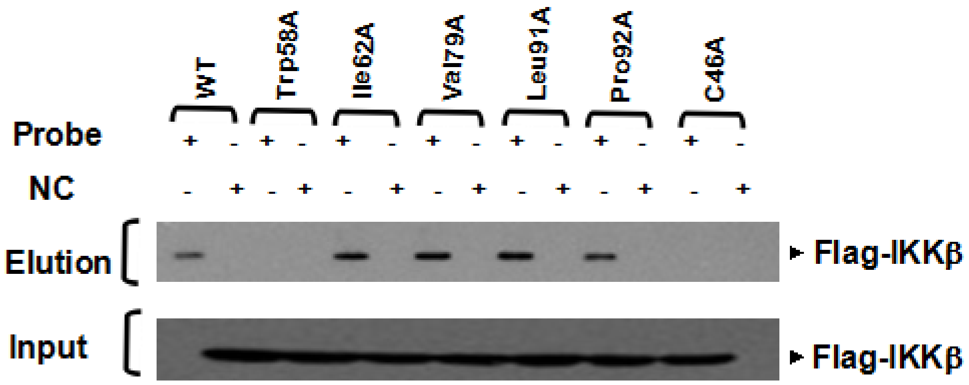

Supplementary Figure 12. Recombinant WT-IKKβ and IKKβ mutations near to Cys46 were incubated with Probe or NC at 37°C for 1.5 h, followed by pull-down with streptavidin-agarose, the precipitates were then resolved by SDS-PAGE and blotted for biotin or flag.

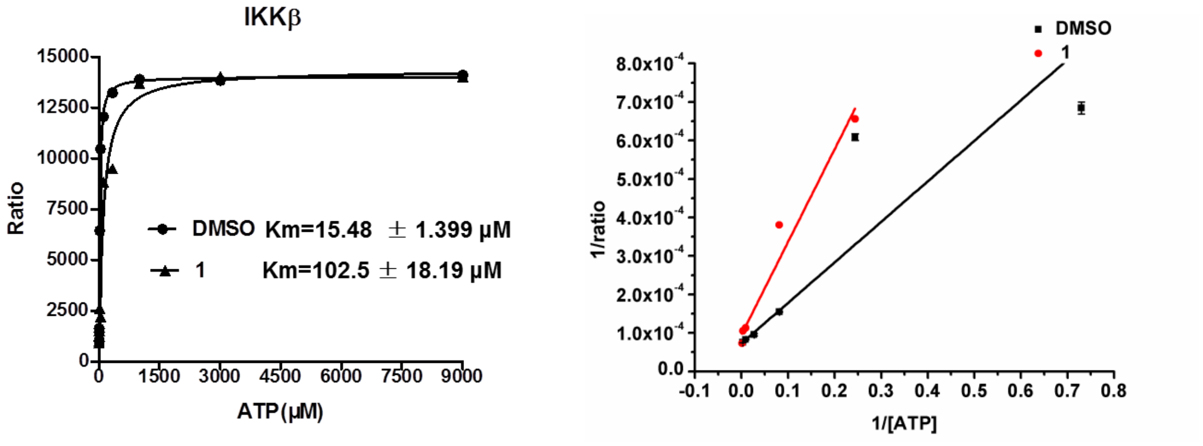

Supplementary Figure 13. Kinetic study of the interaction of ATP with IKKβ in the absence or presence of ainsliadimer A (**1**). Kinetic data were determined at ATP concentrations ranging from 0.15~3000 μM in the presence of ainsliadimer A (**1**) at a concentration of 4 uM. The K_m for ATP was determined with the Michaelis–Menten equation fits in GraphPad Prism 5.0. All experiments were repeated at least three times with the similar results.

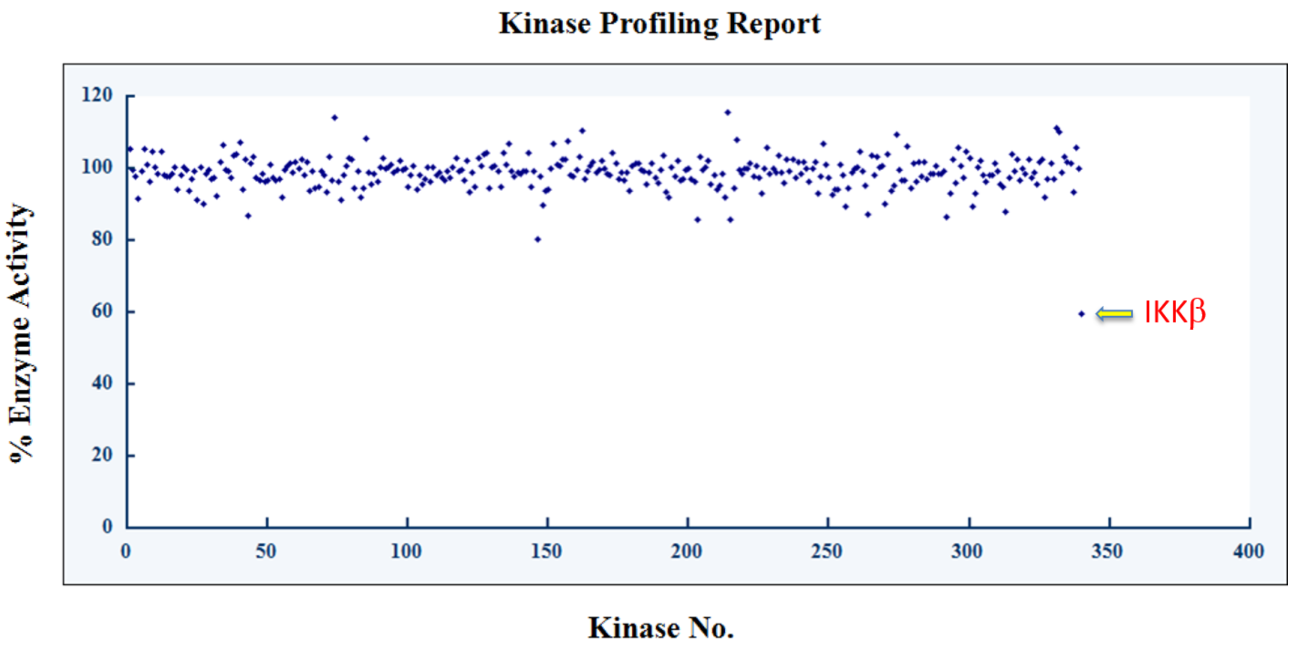

Supplementary Figure 14. The effect of ainsliadimer A (**1**) on a panel of 340 kinases including IKKβ at a concentration of 200 nM. The profiling demonstrates that **1** does not inhibit the enzymatic activity of most of the tested kinases at 200 nM.

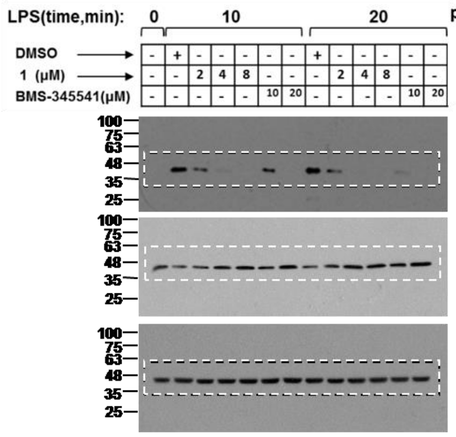

Figure 2a

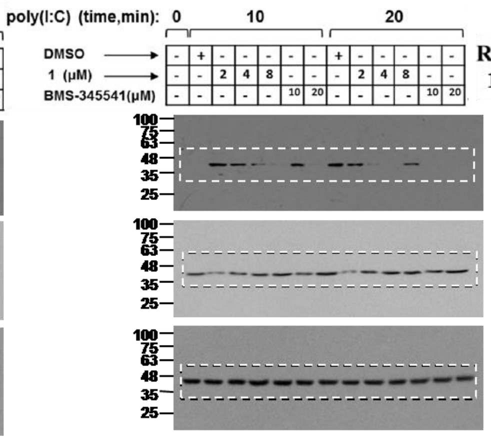

Figure 2b

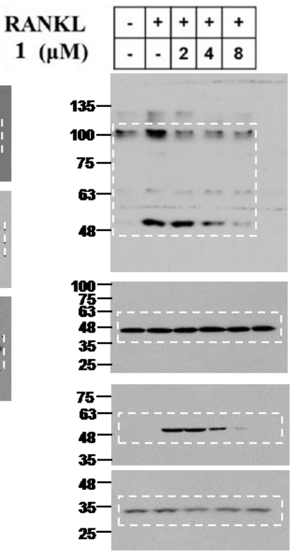

Figure 2f

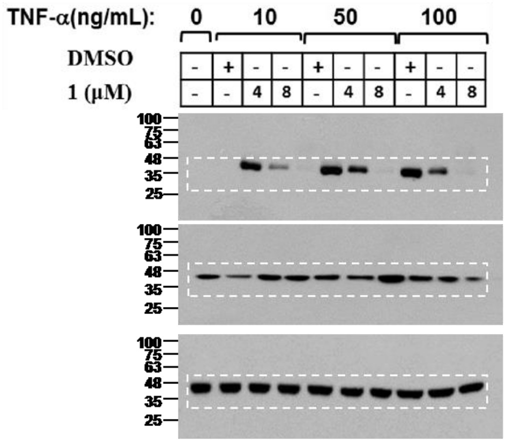

Figure 2c

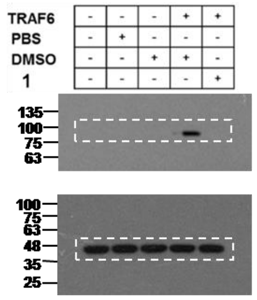

Figure 2e

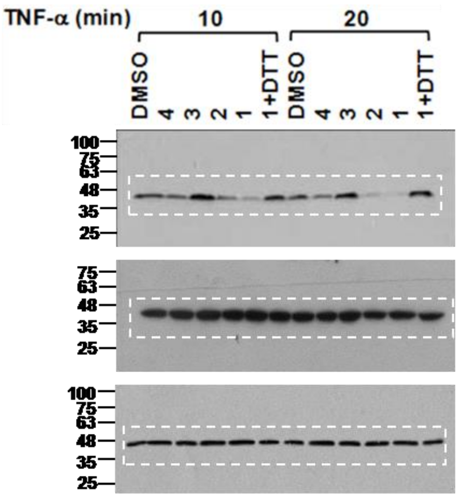

Figure 3b

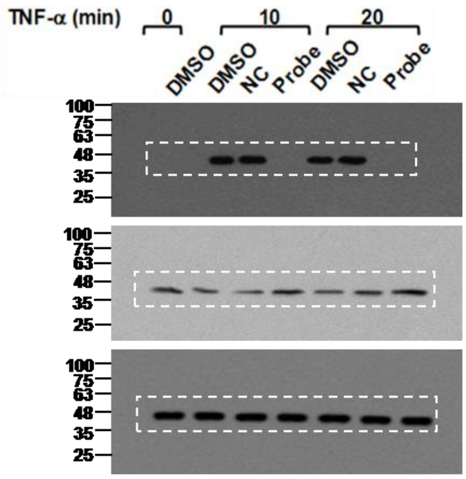

Figure 3d

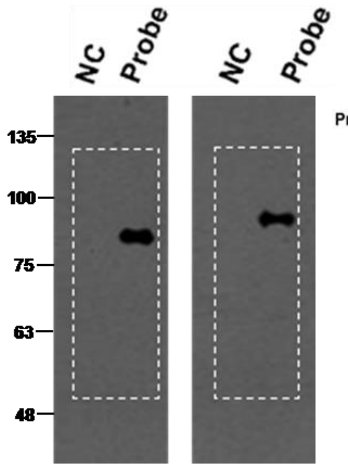

Figure 4b

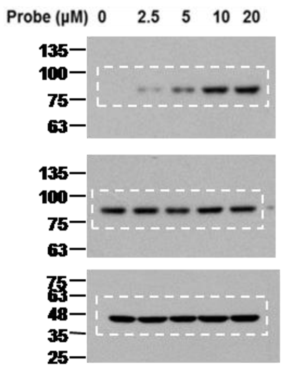

Figure 4c

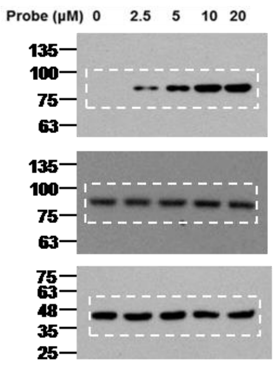

Figure 4d

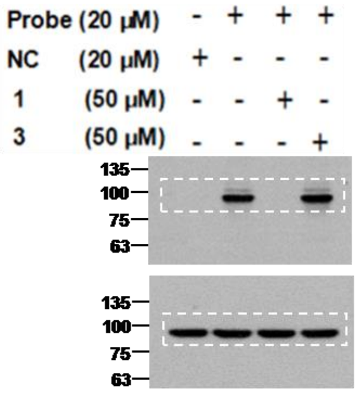

Figure 4e

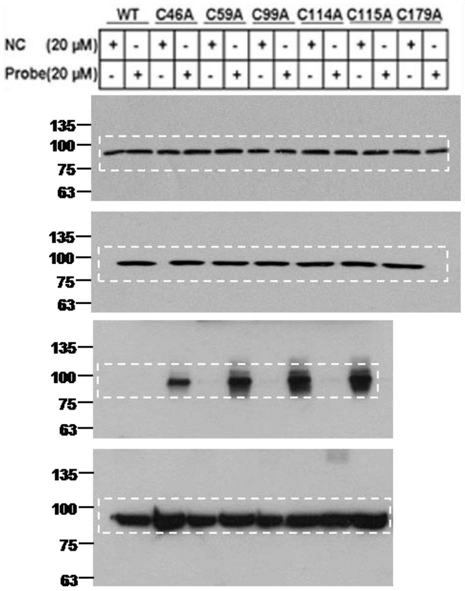

Figure 5a

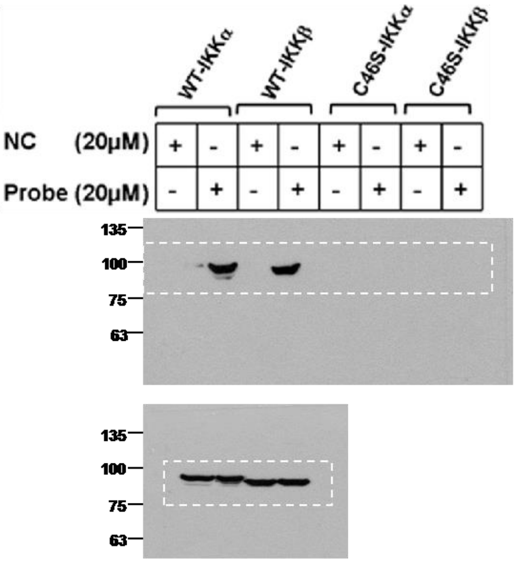

Figure 5b

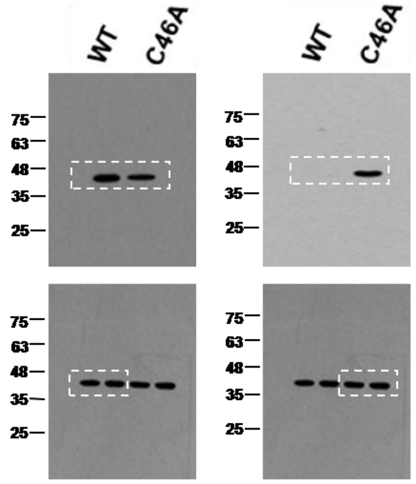

Figure 5d

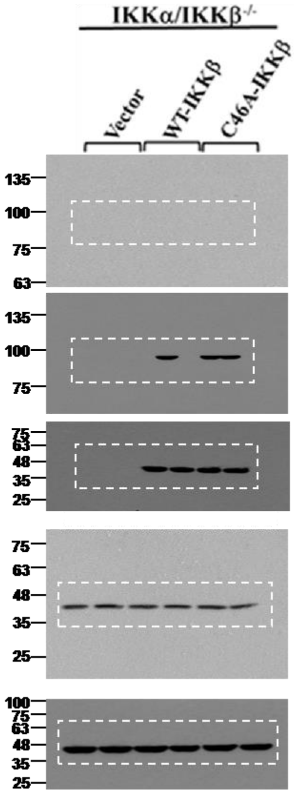

Figure 5e

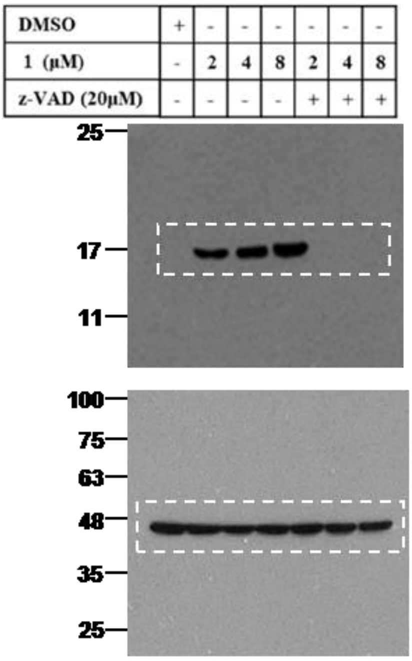

Figure7b

Supplementary Figure 15. Full immunoblots of segments shown in the main figures.

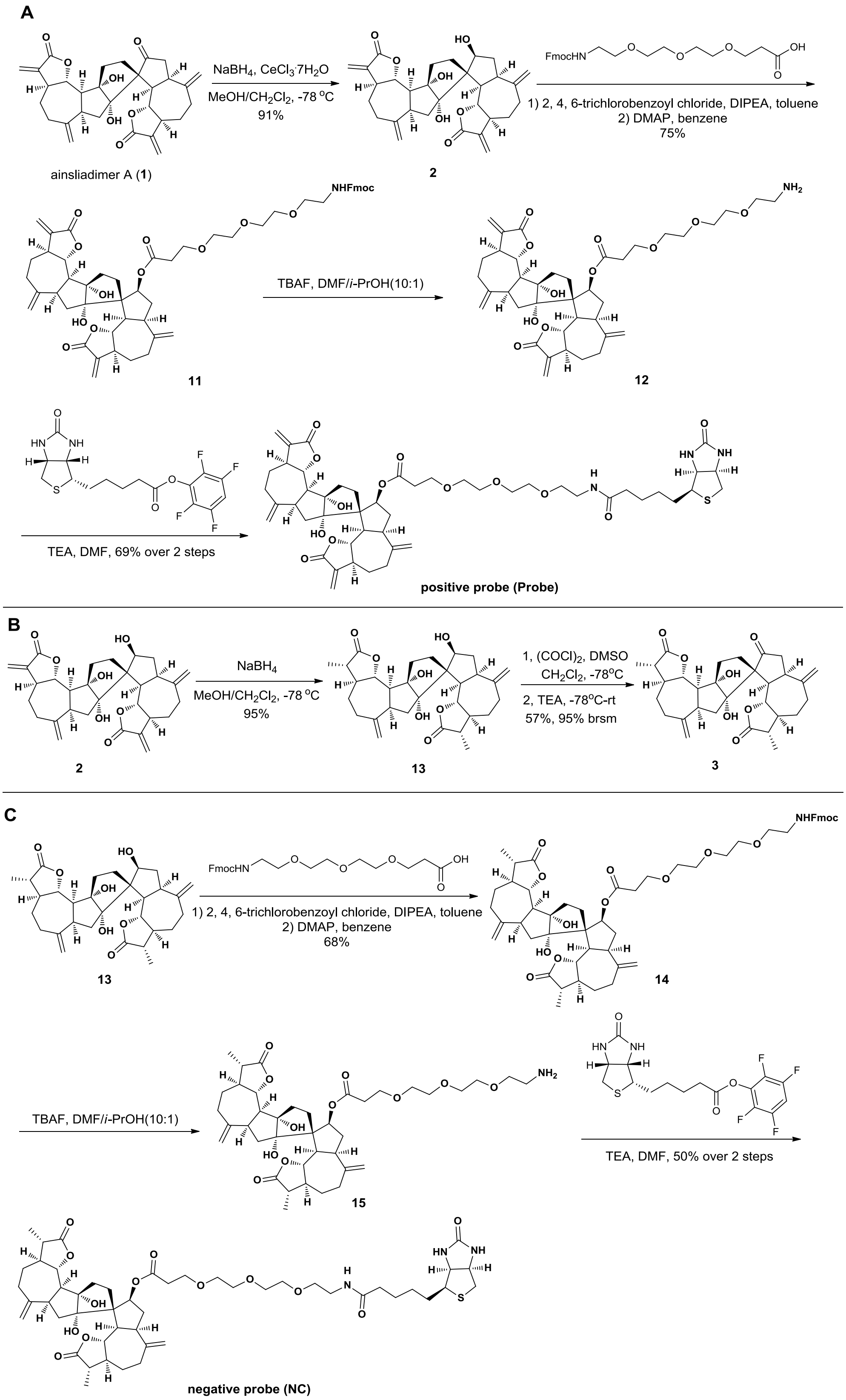

Supplementary Figure 16. Synthetic schemes for positive probe (Probe), inactive analogue **3** and negative probe (NC). A) Synthesis of positive probe (Probe); B) Synthesis of inactive analogue **3**; C) Synthesis of negative probe (NC).

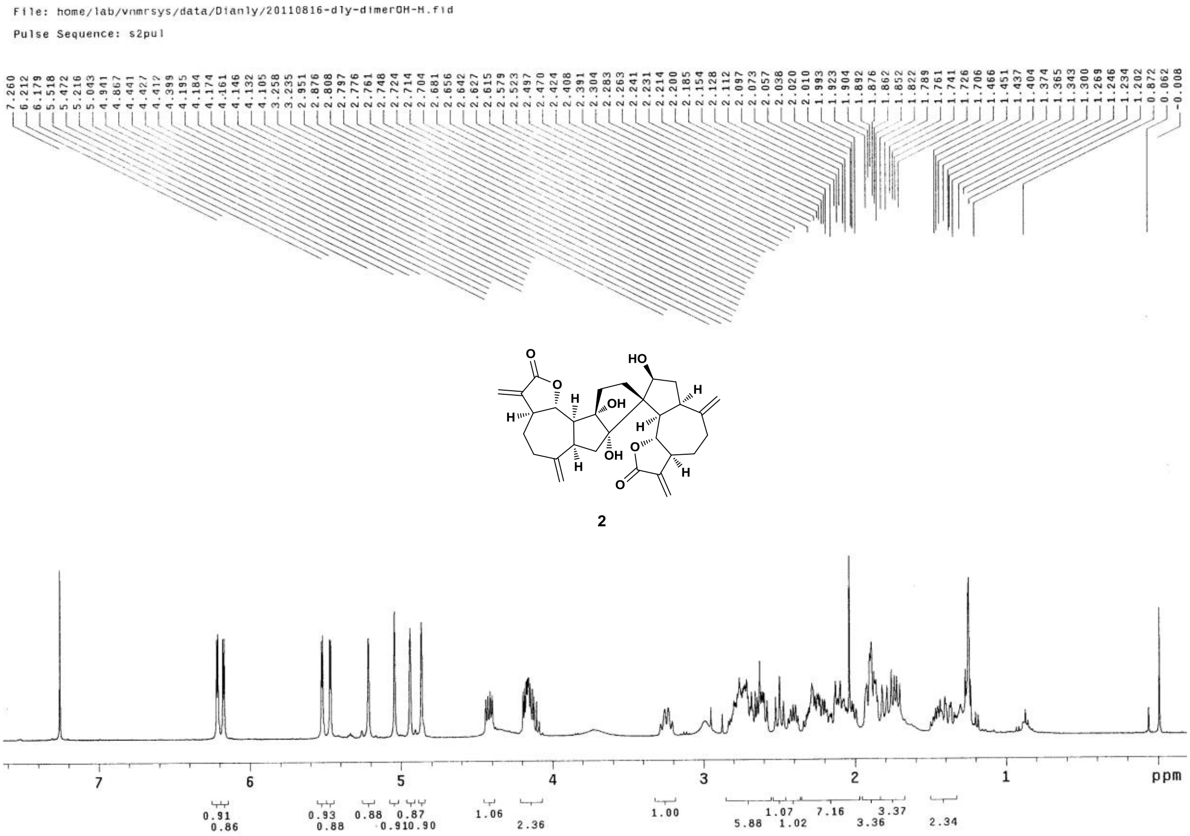

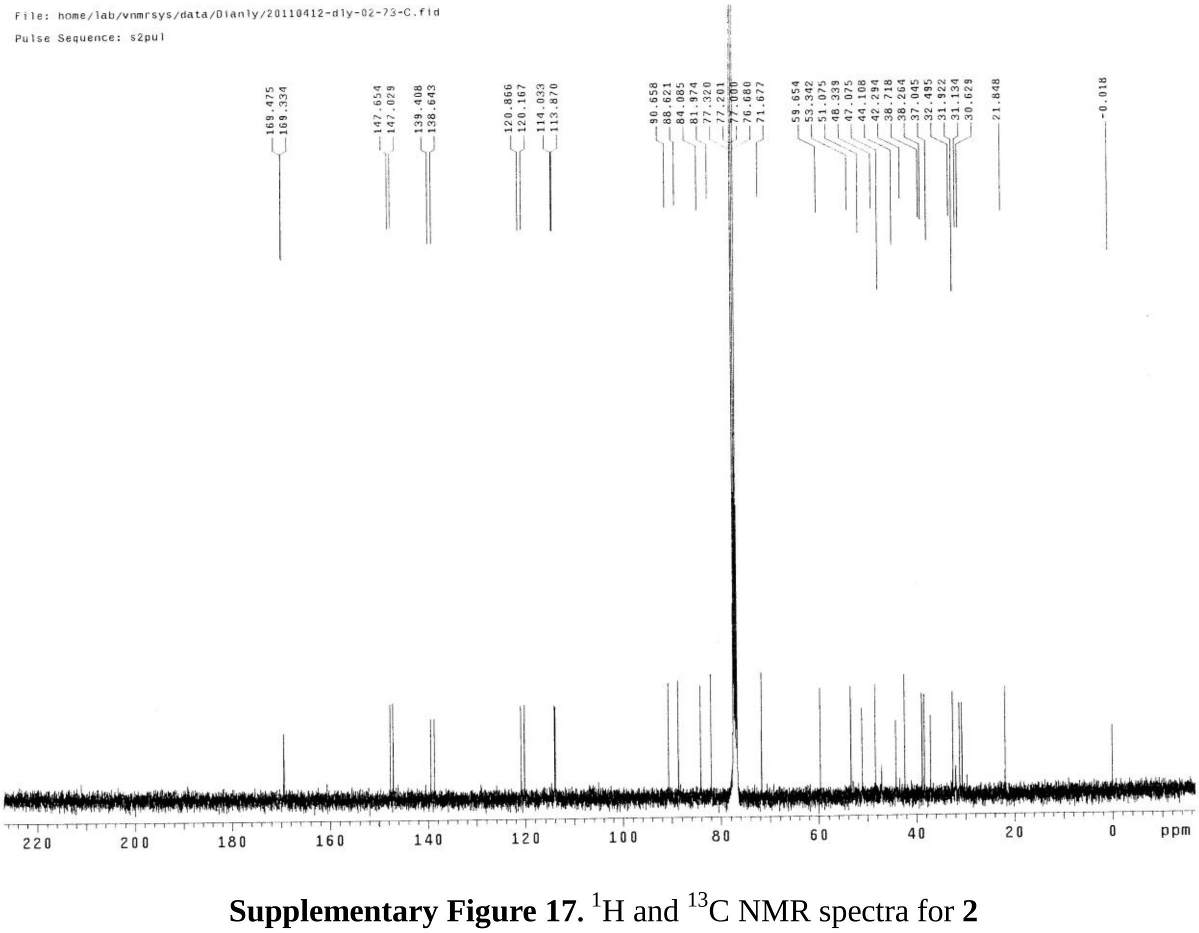

Supplementary Figure 17. ^{1}H and ^{13}C NMR spectra for **2**

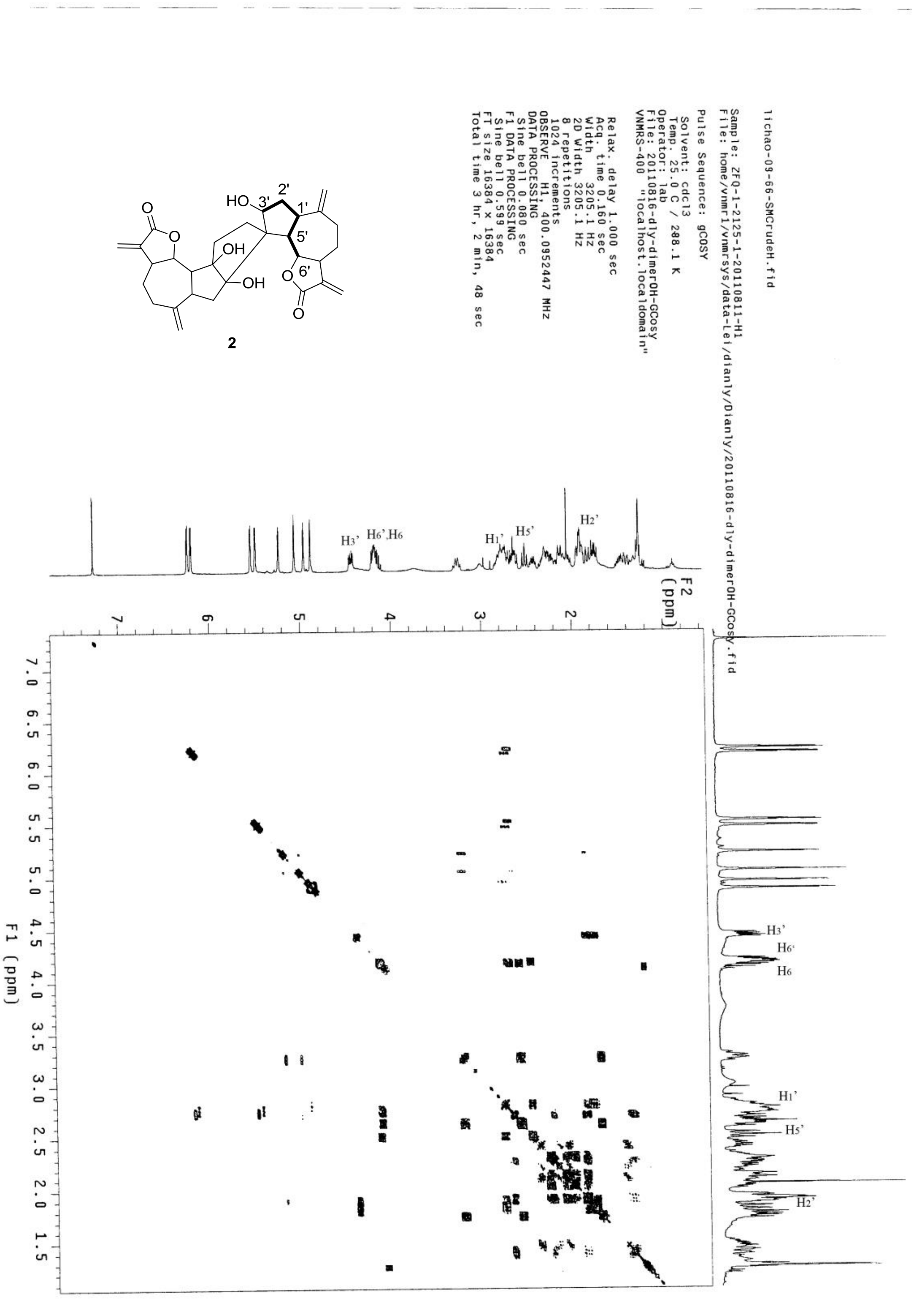

Supplementary Figure 18. ^{1}H-^{1}H COSY spectrum for **2** and the key correlation

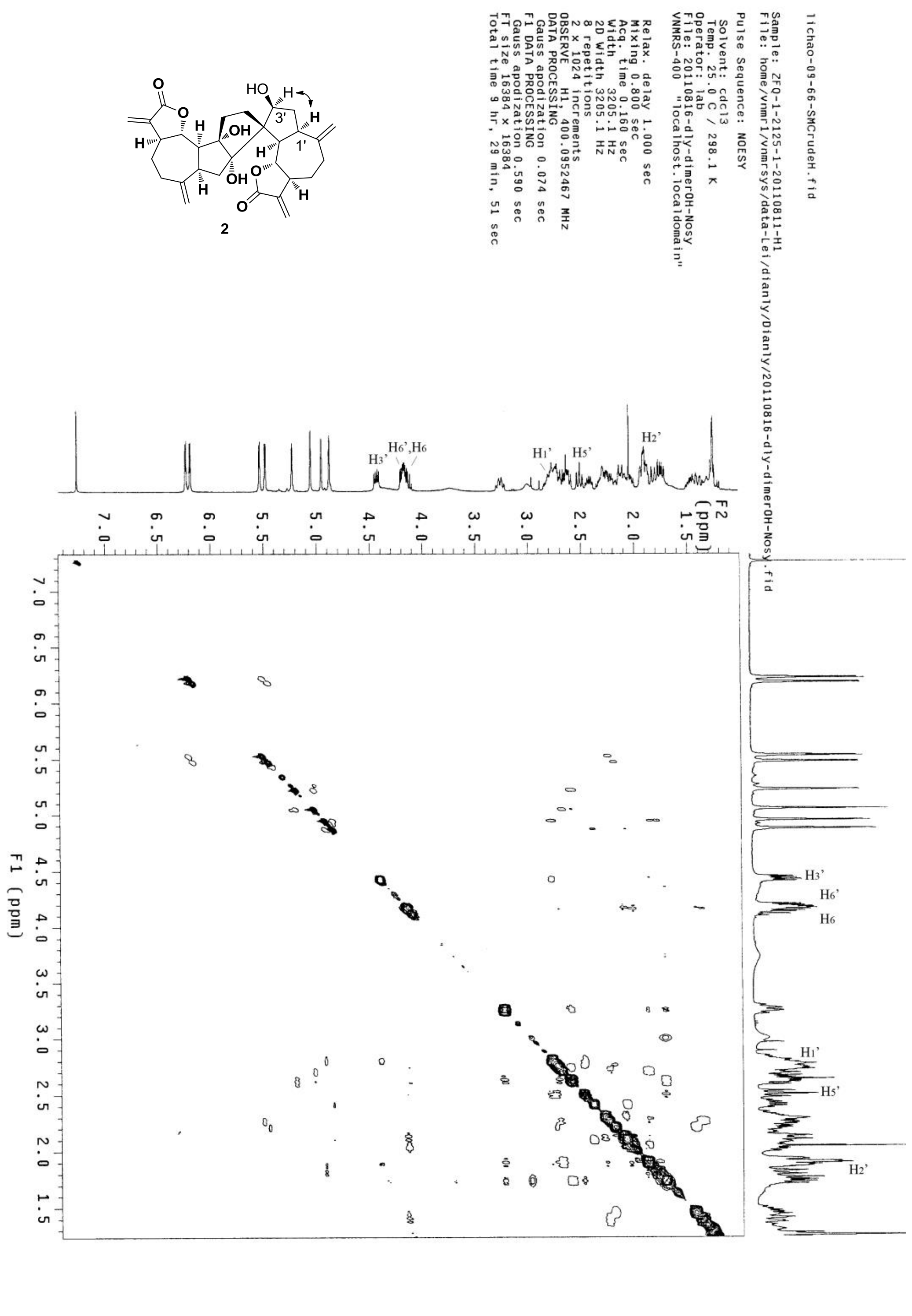

Supplementary Figure 19. NOESY Spectrum for **2** and the key correlation

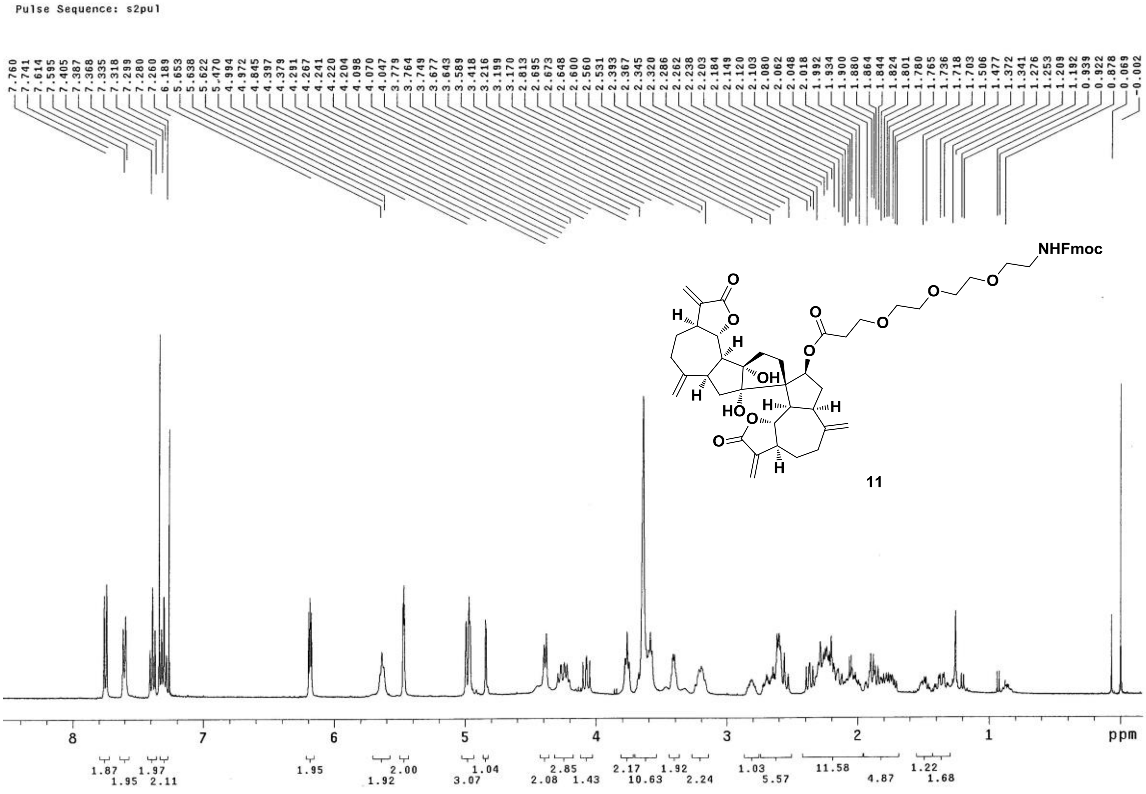

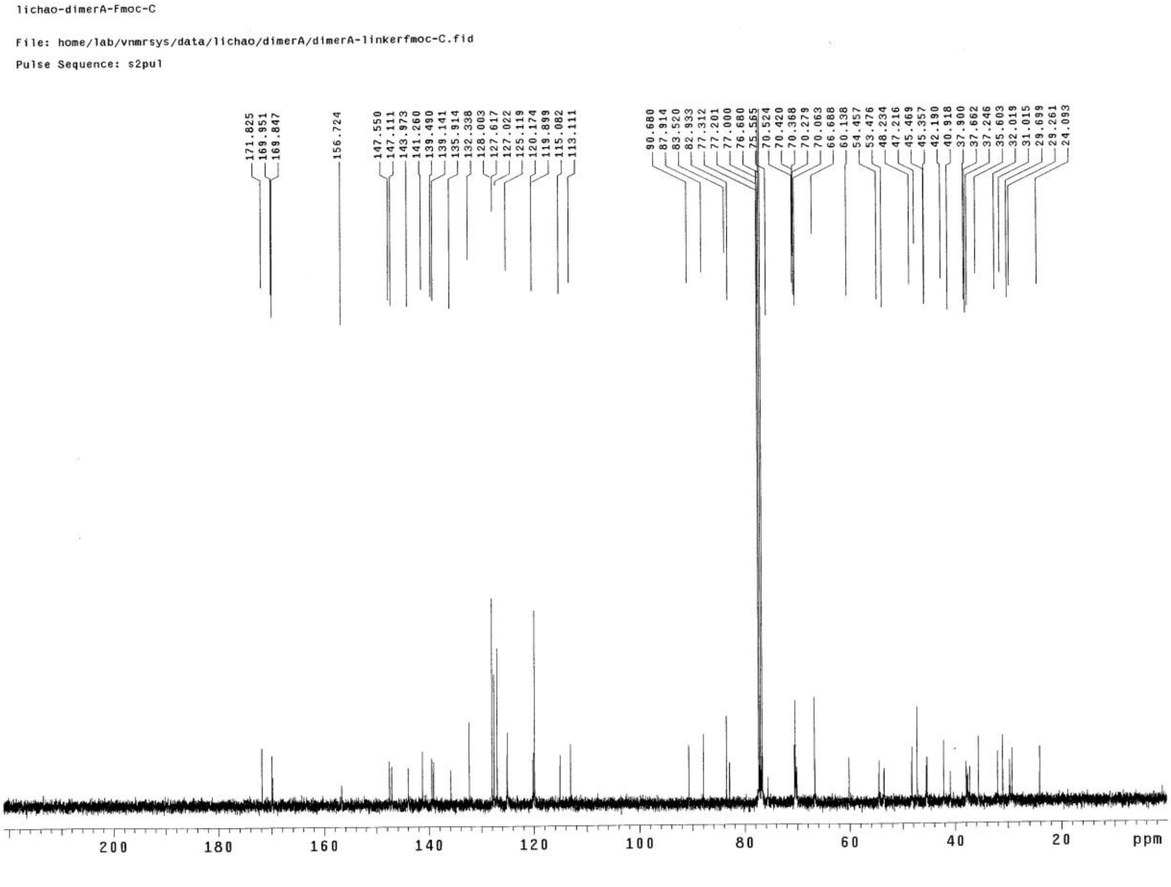

Supplementary Figure 20. ^{1}H and ^{13}C NMR spectra for **11**

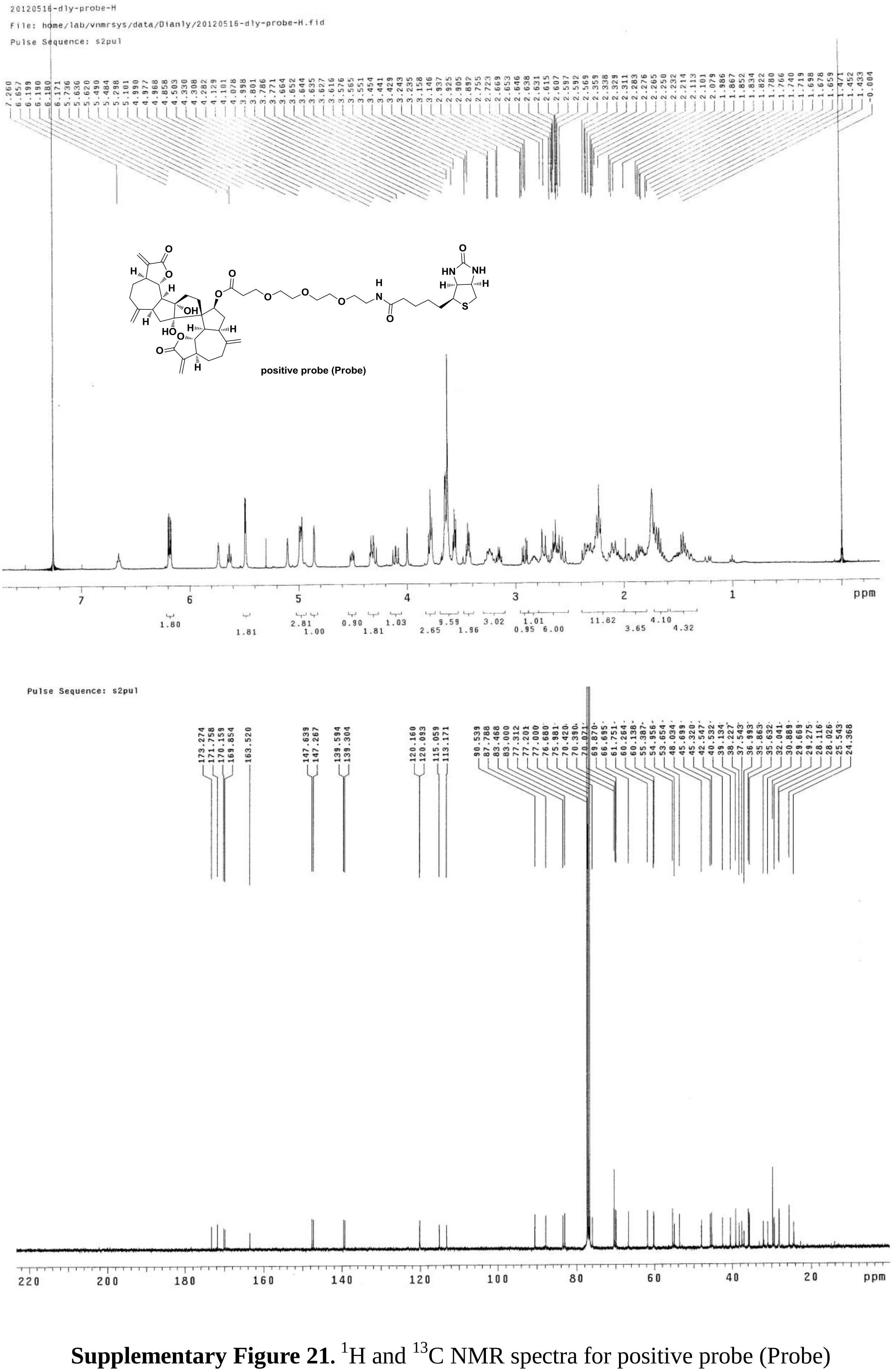

Supplementary Figure 21. ^{1}H and ^{13}C NMR spectra for positive probe (Probe)

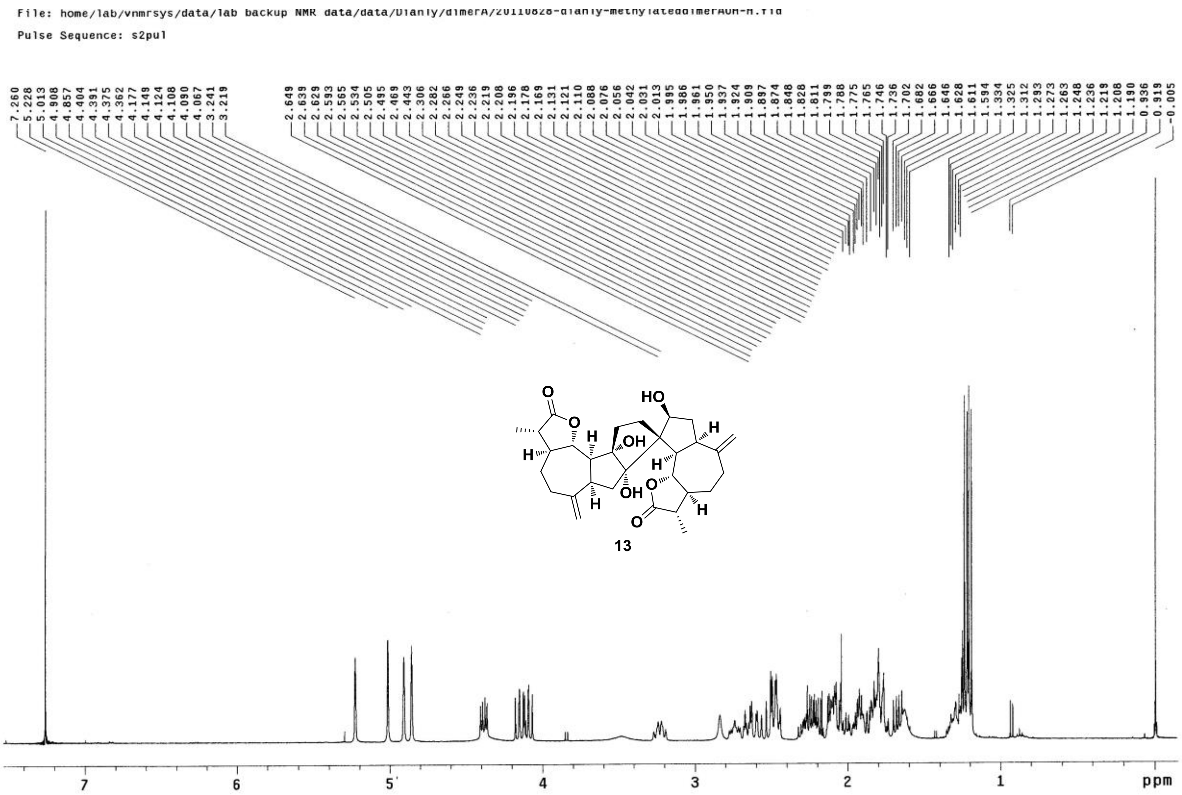

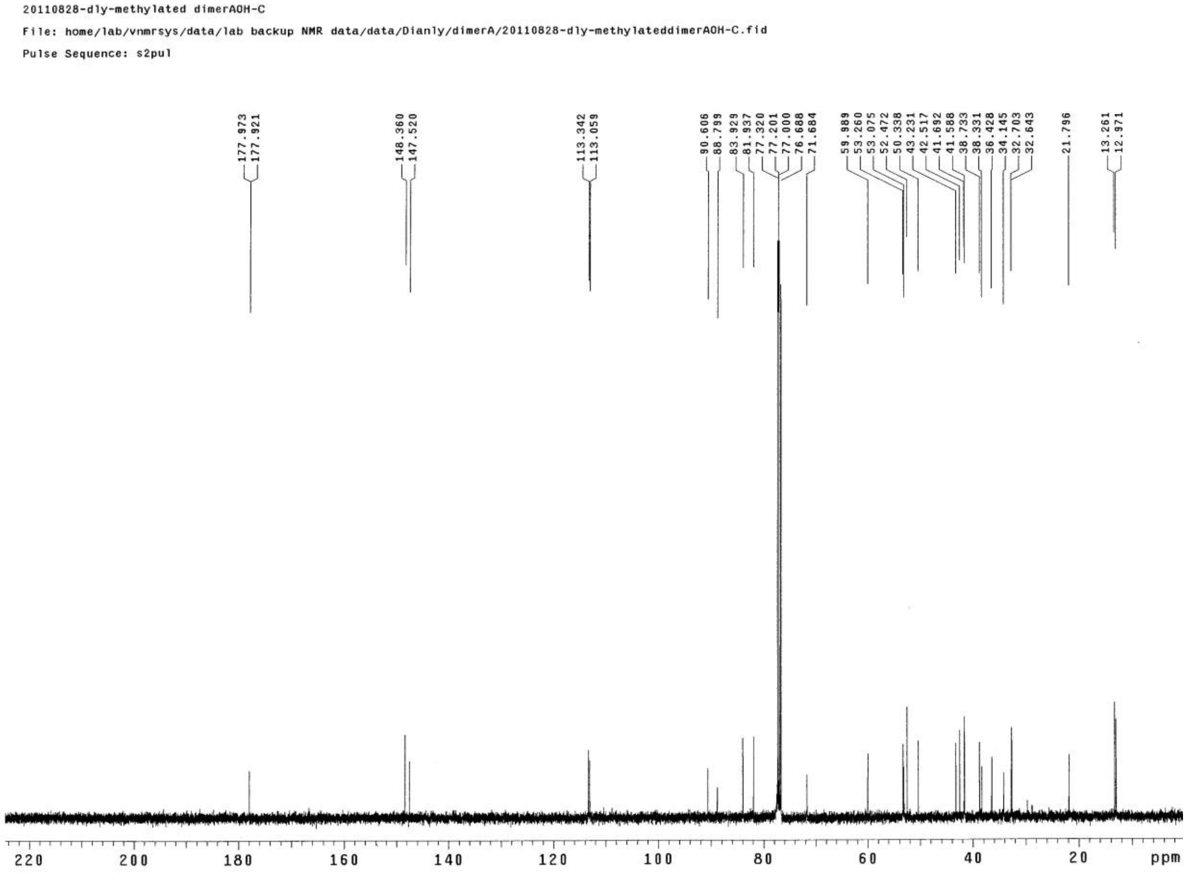

Supplementary Figure 22. ^{1}H and ^{13}C NMR spectra for **13**

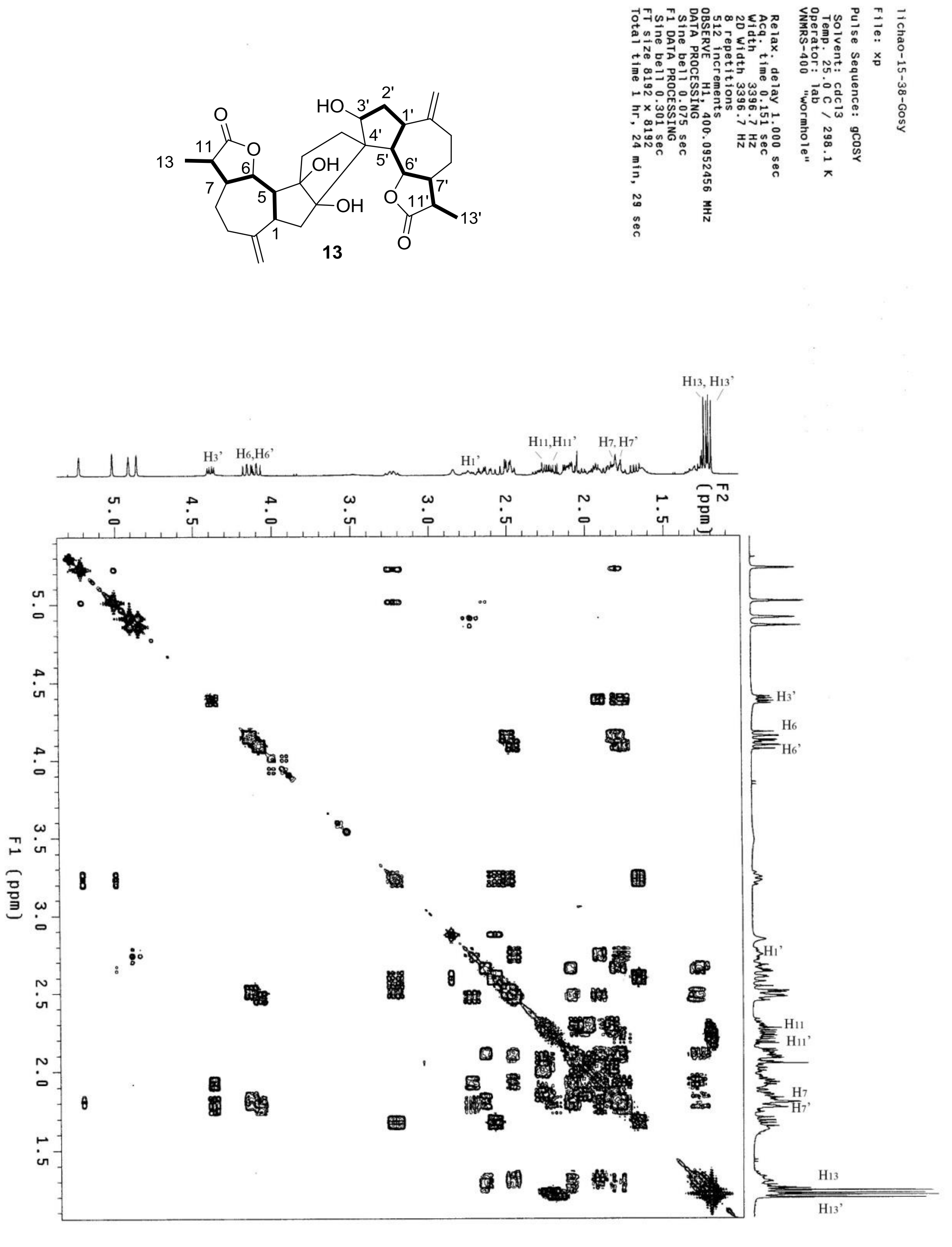

Supplementary Figure 23. ^{1}H-^{1}H COSY spectrum for **13** and the key correlation (-)

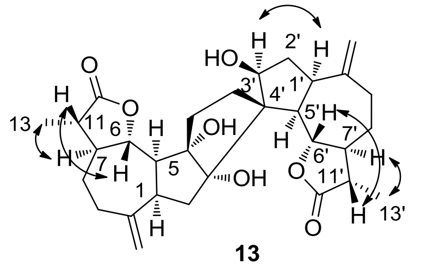

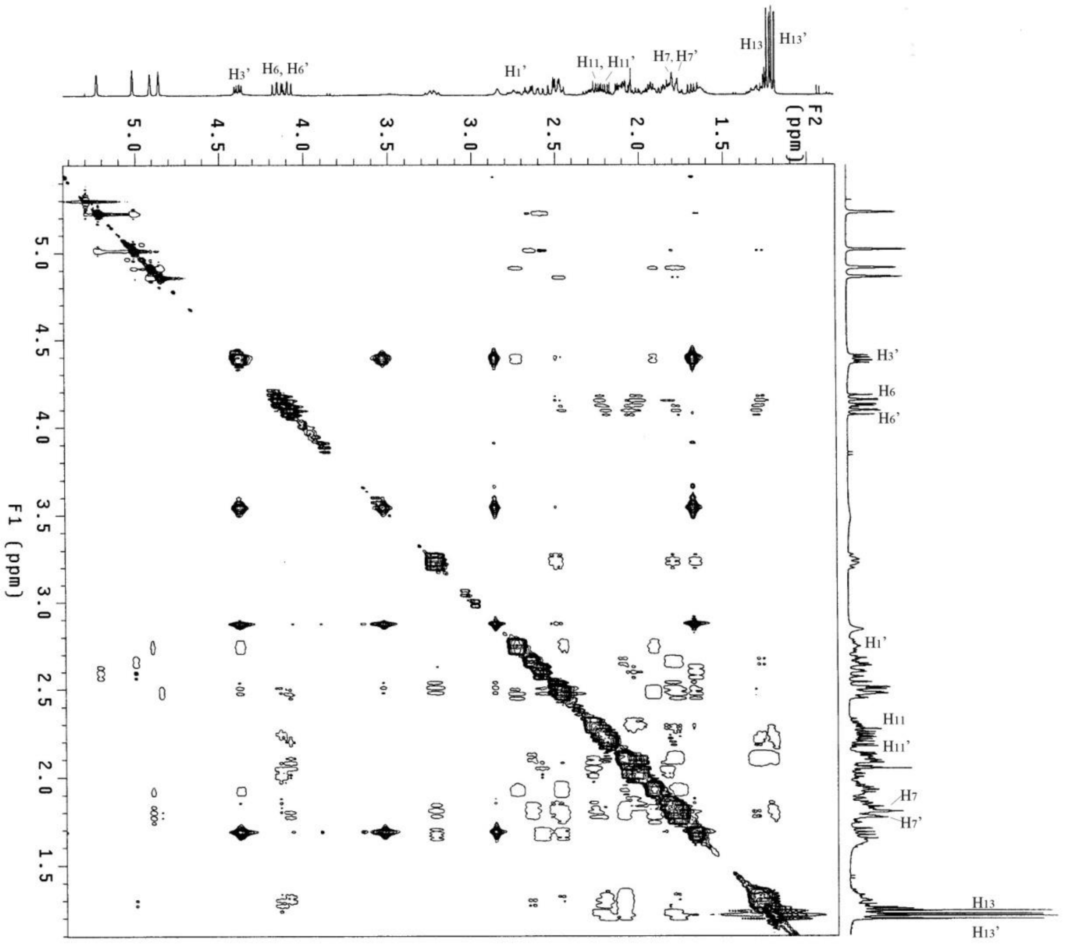

Supplementary Figure 24. NOESY spectrum for **13** and the key correlation

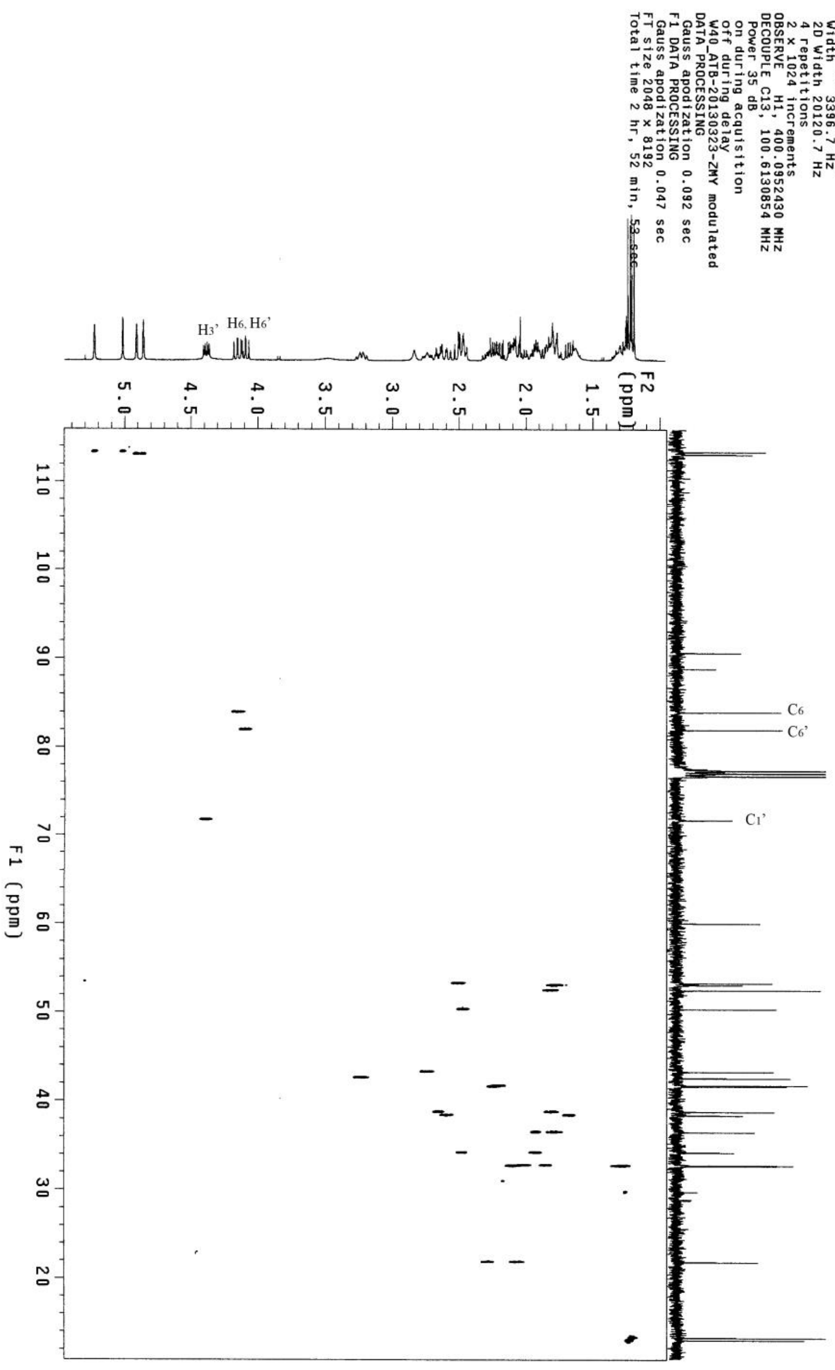

Supplementary Figure 25. HSQC Spectrum for **13**

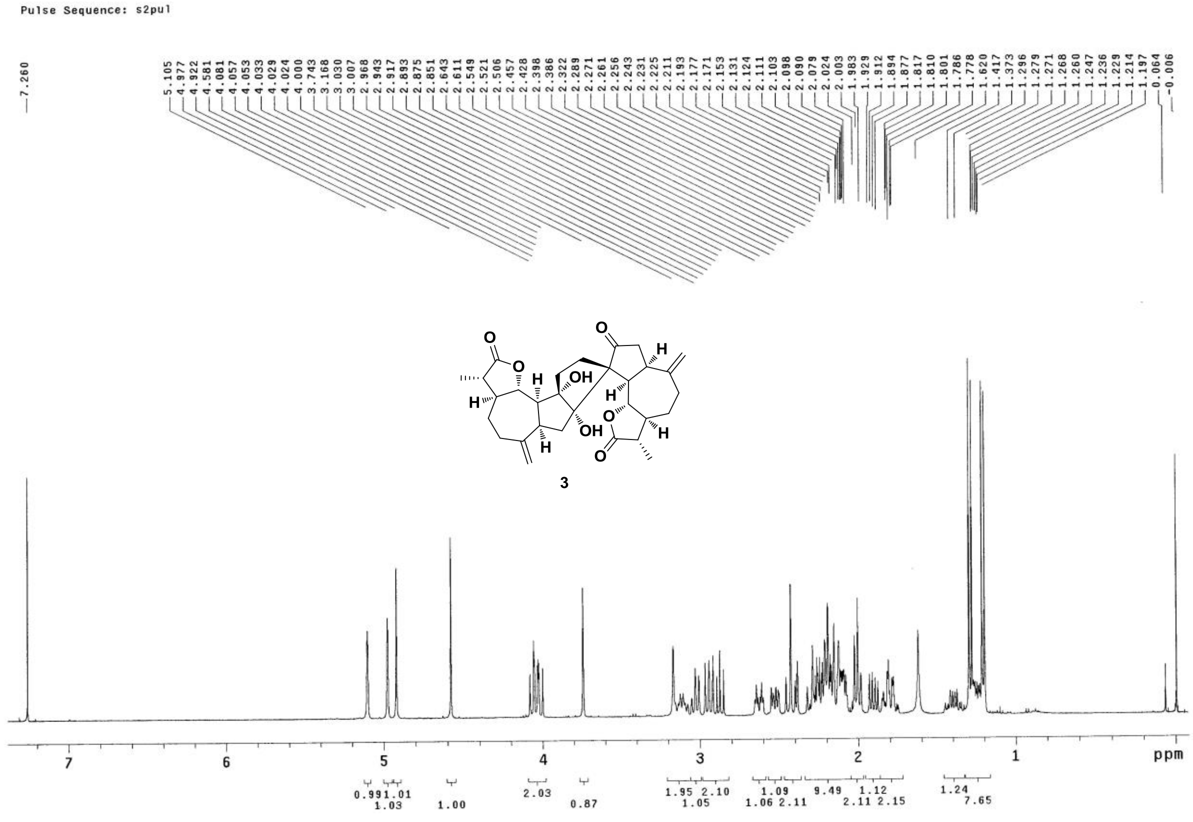

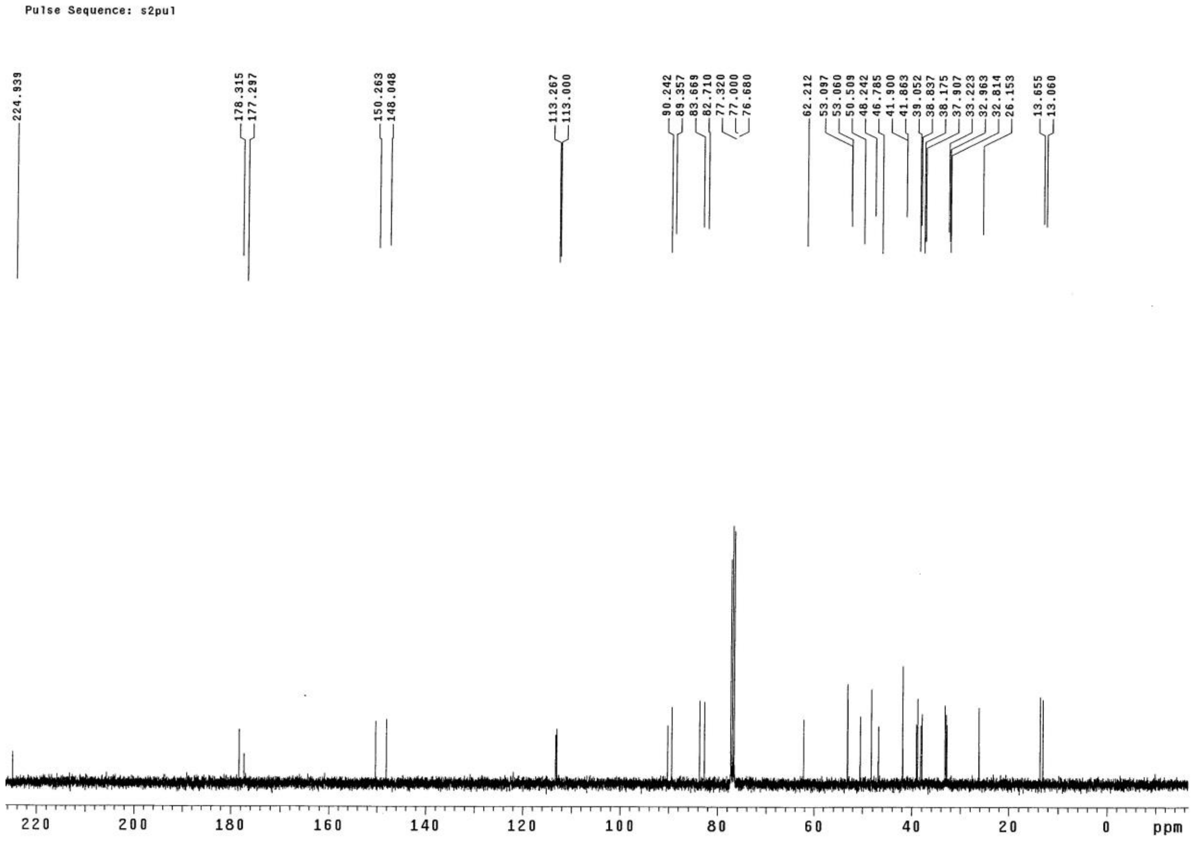

Supplementary Figure 26. ^{1}H and ^{13}C NMR spectra for **3**

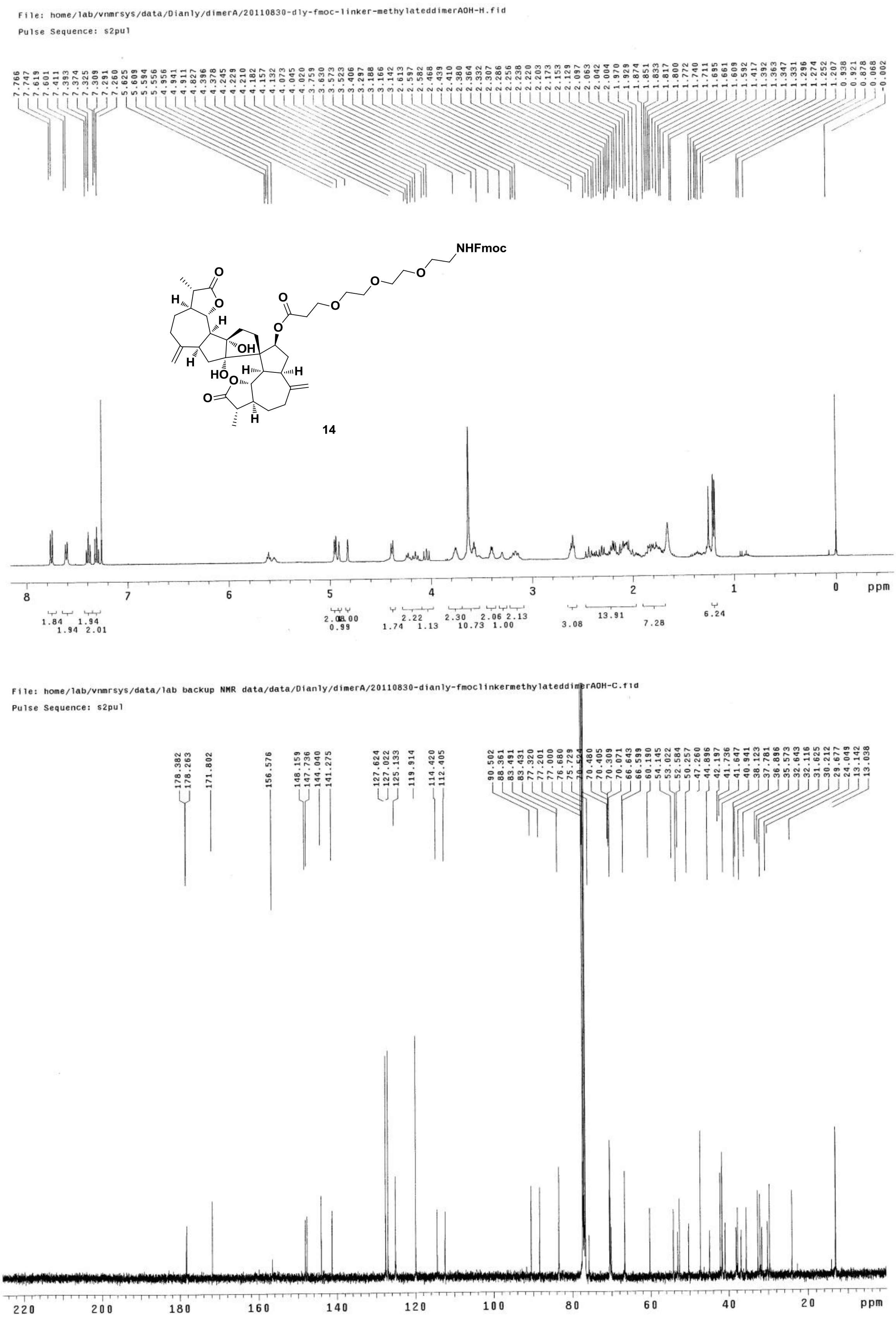

Supplementary Figure 27. ^{1}H and ^{13}C NMR spectra for **14**

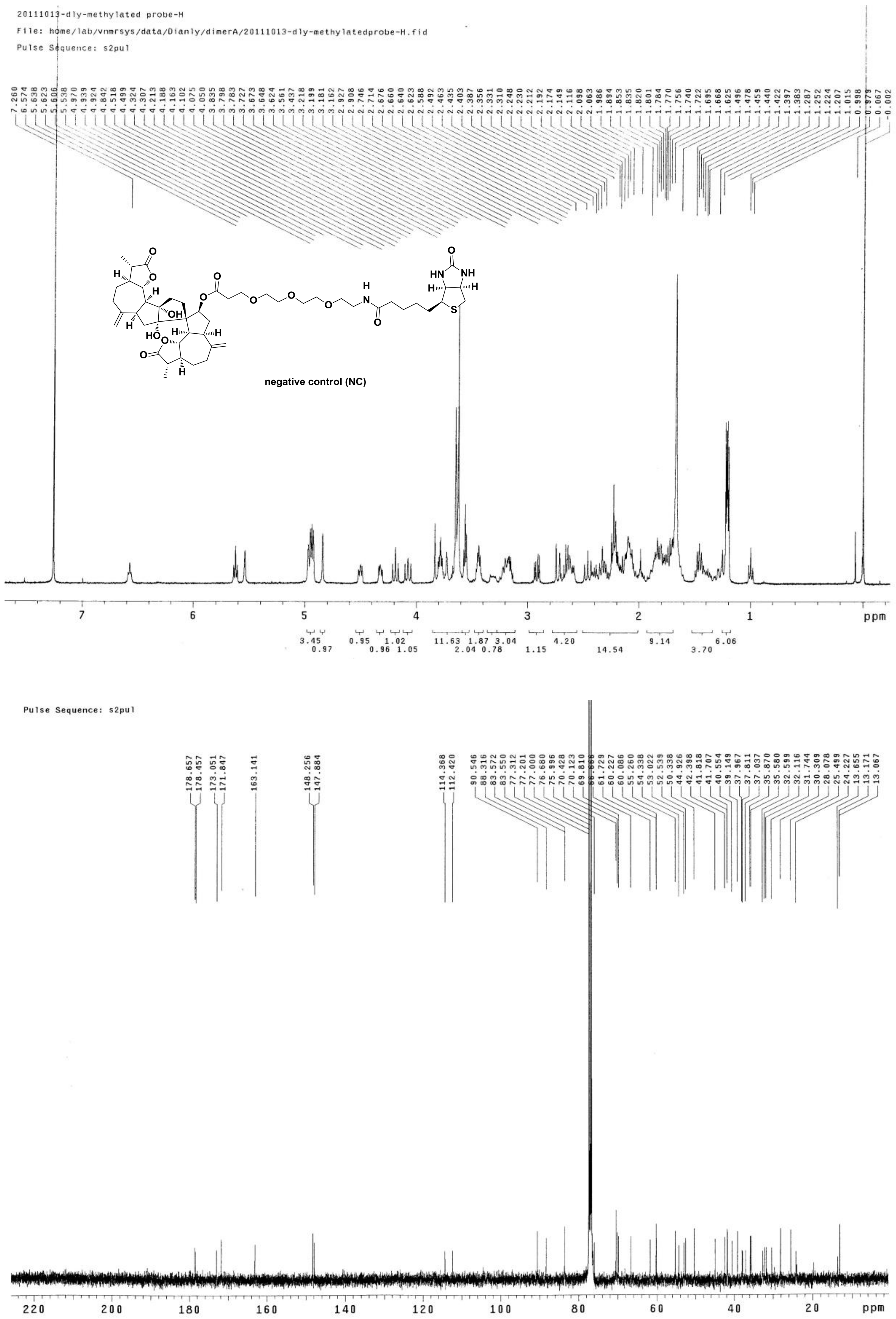

Supplementary Figure 28. ^{1}H and ^{13}C NMR spectra for negative probe (NC)

Supplementary Tables

Supplementary Table 1: primers for IKKα and IKKβ mutation constructs

Mutants	Primers: Forward primers (F), Reverse primers (R)
IKKα	F:5’-AAAATAGCAATTAAGTCTTATCGCCTAGAGCTAAGTACC
(Cys46Ala)	R:3’-AAAATAGCAATTAAGTCTTATCGCCTAGAGCTAAGTACC
IKKα	F:5’-CTCAAAATAGCAATTAAGTCTAGTCGCCTAGAGCTAAGTACC
(Cys46Ser)	R:3’-GGTACTTAGCTCTAGGCGACTAGACTTAATTGCTATTTTGAG
IKKβ	F:5’-GCAGATTGCCATCAAGCAGAGCCGGCAGGAGCTCAGCCCCCGG
(Cys46Ser)	R:3’-CCGGGGGCTGAGCTCCTGCCGGCTCTGCTTGATGGCAATCTGC
IKKβ□□□	F:5’-GCAGATTGCCATCAAGCAGGCCCGGCAGG
(Cys46Ala)	R:3’-GGGGCTGAGCTCCTGCCGGGCCTGCTTGATGGCAATCTGC
IKKβ□□□	F:5’-CGGAACCGAGAGCGGTGGGCCCTGGAGAT
(Cys59Ala)	R:3’-CATGATCTGGATCTCCAGGGCCCACCGCTCTCGGTTCCG
IKKβ□□□	F:5’-CTGCTGGCCATGGAGTACGCCCAAGGAGGAGATCTCCGG
(Cys99Ala)	R:3’-CCGGAGATCTCCTCCTTGGGCGTACTCCATGGCCAGCAG
IKKβ□□□	F:5’-CTGAACCAGTTTGAGAACGCCTGTGGTCTGCGGGAAGGTG
(Cys114Ala)	R:3’-CACCTTCCCGCAGACCACAGGCGTTCTCAAACTGGTTCAG
IKKβ□□□	F:5’-GAACCAGTTTGAGAACTGCGCTGGTCTGCGGGAAGGTGCC
(Cys115Ala)	R:3’-GGCACCTTCCCGCAGACCAGCGCAGTTCTCAAACTGGTTC
IKKβ□□□	F:5’-GATCAGGGCAGTCTTGCCACATCATTCGTGGGGAC
(Cys179Ala)	R:3’-GTCCCCACGAATGATGTGGCAAGACTGCCCTGATC
IKKβ□□□	F:5’-CCTGGCCTTTGAGGCCATCACGGGCTTCCGGCC
(Cys215Ala)	R:3’-GGCCGGAAGCCCGTGATGGCCTCAAAGGCCAGG
IKKβ□□□	F:5’-GCAGCTCAGTAAAACTGTGGTTGCCAAGCAGAAGGC
(Cys618Ala)	R:3’-GTTCCAGCGCCTTCTGCTTGGCAACCACAGTTTTACTGAGCTGC
IKKβ□□□	F:5’-GGAATCTCCTGAAGATTGCTGCTAGCAAGGTCCGTGG
(Cys662Ala)	R:3’-GACAGGACCACGGACCTTGCTAGCAGCAATCTTCAGGAGATTC

Supplementary Table 2: Primer sequences for RT-qPCR

Primer name	sequence
GAPDH-F	5’- GTGTTCCTACCCCCAATGTGT-3’
GAPDH-R	5’- ATTGTCATACCAGGAAATGAGCTT-3’
c-FLIP-F	5’- GAAAGAGGTAAGCTGTCTGTCG-3’
c-FLIP-R	5’- CGACAGACAGCTTACCTCTTTC-3’
c-IAP1-F	5’- AGCACGATCTTGTCAGATTGG-3’
c-IAP1-R	5’- GGCGGGGAAAGTTGAATATGTA-3’
Bcl-xL-F	5’-GATCCCCATGGCAGCAGTAAAGCAAG-3’
Bcl-xL-R	5’-CCCCATCCCGGAAGAGTTCATTCACT-3’

Supplementary Notes

Supplementary Note 1. ^{1}H NMR spectra were recorded on a Varian 400 MHz spectrometer at ambient temperature with $CDCl_3$ as the solvent unless otherwise stated. ^{13}C NMR spectra were recorded on a Varian 100 MHz spectrometer (with complete proton decoupling) at ambient temperature. Chemical shifts are reported in parts per million relative to chloroform (^{1}H, δ 7.26; ^{13}C, δ 77.00). Data for ^{1}H NMR are reported as follows: chemical shift, multiplicity (s = singlet, d = doublet, t = triplet, q = quartet, m = multiplet, *br* = broad), coupling constants and integration. Infrared spectra were recorded on a Thermo Fisher FT-IR200 spectrophotometer. High-resolution mass spectra were obtained at Peking University Mass Spectrometry Laboratory using a Bruker APEX Flash chromatography. Optical rotations were recorded on an AUTOPOL III digital polarimeter at 589 nm and are recorded as $[\alpha]_D^{20}$ (concentration in grams/100 mL solvent). The samples were analyzed by HPLC-MS on a Waters Auto Purification LC/MS system (3100 Mass Detector, 2545 Binary Gradient Module, 2767 Sample Manager, and 2998 Photodiode Array (PDA) Detector). The system was equipped with a Waters C18 5 μm X-bridge separation column (150*4.6 mm), equilibrated with HPLC grade water (solvent A) and HPLC grade methanol (solvent B) with a flow rate of 1.0 mL/min at room temperature. Preparative HPLC-MS on a Waters Auto Purification LC/MS system (3100 Mass Detector, 2545 Binary Gradient Module, 515 HPLC pump, 2767 Sample Manager, and 2998 Photodiode Array (PDA) Detector). The system was equipped with a Waters C18 5 μm X-bridge separation column (150*19 mm). Thin layer chromatography was performed using 0.25 mm silica gel 60-F plates. Flash chromatography was performed using 200-400 mesh silica gel. Yields refer to chromatographically and spectroscopically pure materials, unless otherwise stated.

Supplementary Note 2. All chemical reagents were used as supplied by Sigma-Aldrich, J&K and Alfa Aesar Chemicals. Methylene chloride, toluene, benzene, DMF were distilled from calcium hydride. All reactions were carried out in oven-dried glassware under an argon atmosphere unless otherwise noted.

Supplementary Methods

Synthesis of 2 from natural product (+)-ainsliadimer A (1)

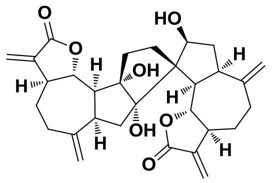

2: To a solution of (+)-ainsliadimer A (**1**, 83.0 mg, 0.164 mmol) in MeOH (11.0 mL) and DCM (2.7 mL) was added $CeCl_3 \cdot 7H_2O$ (74 mg, 0.199 mmol). After stirred at room temperature for 15 min, the mixture was cooled to -78 ^oC. To this cooled solution was added a freshly prepared solution of $NaBH_4$ (7.5 mg, 0.199 mmol) in MeOH (2.7 mL). After stirred at this temperature for 1 h, the reaction mixture was quenched by acetone (5 mL), and poured into sat. aqueous NH_4Cl (20 mL). The resulting mixture was extracted with EtOAc (30 mL×3). The combined organic layers were washed with brine, dried over anhydrous sodium sulfate, and concentrated in vacuo. The residue was purified by flash chromatography (silica gel, CH_2Cl_2:MeOH = 25:1) to afford **2** (76.0 mg, 91 %) as a colorless oil.

2: TLC (CH_2Cl_2:MeOH, 20:1 v/v): R_f = 0.40; $[\alpha]^{24}_D$ -17.0 (*c* 0.83, CH_2Cl_2); ^{1}H NMR (400 MHz, $CDCl_3$) δ 1.34-1.47 (m, 2H), 1.71-1.92 (m, 6H), 1.99-2.30 (m, 7H), 2.39-2.42 (m, 1H), 2.50 (t, *J* = 10.4 Hz, 1H), 2.57-2.88 (m, 6H), 2.95 (s, *br*, 1H), 3.25 (q, *J* = 9.2 Hz, 1H), 4.11-4.20 (m, 2H), 4.42 (dd, *J* = 5.6 Hz, 11.6 Hz, 1 H), 4.87 (s, 1H), 4.94 (s, 1H), 5.04 (s, 1H), 5.22 (s, 1H), 5.47 (d, *J* = 2.8 Hz, 1H), 5.52 (d, *J* = 2.8 Hz, 1H), 6.18 (d, *J* = 3.6 Hz, 1H), 6.21 (d, *J* = 3.6 Hz, 1H); ^{13}C NMR (100 MHz, $CDCl_3$) δ 21.8, 30.6, 31.1, 31.9, 32.5, 37.0, 38.3, 38.7, 42.3, 44.1, 47.1, 48.3, 51.1, 53.3, 59.7, 71.7, 82.0, 84.1, 88.6, 90.7, 113.9, 114.0, 120.2, 120.9, 138.6, 139.4, 147.0, 147.7, 169.3, 169.5; IR (neat) ν_{max} 3412, 2932, 2861, 1762, 1664, 1637, 1445, 1406, 1302, 1262, 1146, 998, 963, 902 cm^{-1}; HRMS (ESI) [M + H^+] calculated for $C_{30}H_{37}O_7$: 509.2534, found: 509.2534.

Preparation of Probe from 2

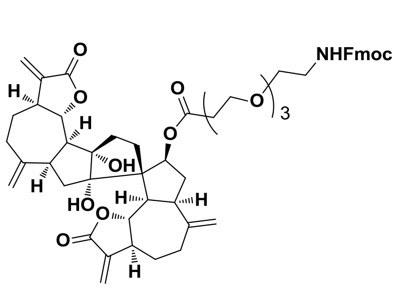

11: To a solution of Fmoc-12-amino-4, 7, 10- trioxadodecanoic acid[i] (163 mg, 0.368 mmol) in toluene (4.5 mL) was added 2, 4, 6-trichlorobenzoyl chloride (343 μL, 2.12 mmol) and DIPEA (463 μL, 2.66 mmol). After stirred at room temperature for 45 min, the reaction mixture was concentrated in vacuo. The residue was dissolved in benzene (18.0 mL) and added to **2** (90 mg, 0.177 mmol). To the resulting mixture was added a solution of DMAP (259 mg, 2.12 mmol) in benzene (9.0 mL) over 20

min. The reaction mixture was stirred at room temperature for 1.5 h before poured into a mixture of 0.5 M aqueous HCl (50 mL) and EtOAc (20 mL). The organic layer was separated and the aqueous layer was extracted with EtOAc (50 mL×2). The combined organic layers were washed with sat. aqueous $NaHCO_3$, brine sequentially, and dried over anhydrous sodium sulfate, concentrated in vacuo. The residue was purified by flash chromatography (silica gel, EtOAc:PE = 2:1) to afford **11** (124 mg, 75 %) as a colorless oil.

11: TLC ($CHCl_3$:MeOH, 30:1 v/v): R_f = 0.50; $[\alpha]^{23}_D$ -5.6 (*c* 0.78, CH_2Cl_2); ^{1}H NMR (400 MHz, $CDCl_3$) δ 1.32-1.41 (m, 1H), 1.46-1.54 (m, 1H), 1.72-1.93 (m, 5H), 1.98-2.32 (m, 10H), 2.37 (t, *J* = 10.8 Hz, 1H), 2.56-2.78 (m, 5H), 2.79-2.85 (m, 1H), 3.17-3.25 (m, 2H), 3.42-3.48 (m, 2H), 3.59-3.84 (m, 10H), 3.76-3.80 (m, 2H), 4.07 (t, *J* = 9.2 Hz, 1H), 4.22-4.29 (m, 2H), 4.38 (d, *J* = 7.2 Hz, 2H), 4.84 (s, 1H), 4.97 (s, 2H), 4.99 (s, 1H), 5.47 (d, *J* = 3.2 Hz, 1H), 5.47 (d, *J* = 2.8 Hz, 1H), 5.64 (t, *J* = 6.2 Hz, 2H), 6.18 (d, *J* = 3.6 Hz, 1H), 6.19 (d, *J* = 3.6 Hz, 1H), 7.32 (t, *J* = 7.2 Hz, 2H), 7. 39 (t, *J* = 7.2 Hz, 2H), 7.60 (d, *J* = 7.6 Hz, 2H), 7.75 (d, *J* = 7.6 Hz, 2H); ^{13}C NMR (100 MHz, $CDCl_3$) δ 24.1, 29.3, 29.7, 31.0, 32.0, 35.6, 37.2, 37.7, 37.9, 40.9, 42.2, 45.4, 45.5, 47.2, 48.2, 53.5, 54.5, 60.1, 66.7, 70.1, 70.3, 70.4, 70.4, 70.5, 75.6, 82.9, 83.5, 87.9, 90.7, 113.1, 115.1, 119.9, 120.2, 120.2, 125.1, 127.0, 127.6, 128.0, 132.3, 135.9, 139.1, 139.5, 141.3, 144.0, 147.1, 147.6, 156.7, 169.8, 170.0, 171.8; IR (neat) ν_{max} 3439, 3070, 2930, 2873, 1766, 1731, 1580, 1547, 1450, 1404, 1351, 1263, 1186, 1145, 1126, 994 cm^{-1}; HRMS (ESI) [M + Na$^+$] calculated for $C_{54}H_{63}NNaO_{13}$: 956.4192, found: 956.4167.

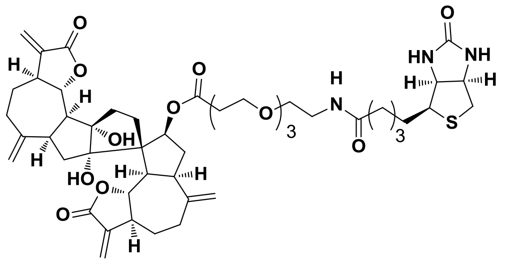

Positive probe (Probe): To a solution of **11** (122 mg, 0.131 mmol) in DMF/*i*-PrOH (10:1, 2.4 mL) was added TBAF (4.9 mL, 0.196 mmol, 0.04 M in DMF). The reaction mixture was stirred at room temperature for 40 min before poured into the mixture of EtOAc (50 mL) and brine (50 mL). The aqueous layer was separated, and the organic layer was washed with brine (50 mL×2). The aqueous layers were further extracted with EtOAc (50 mL) sequentially. The combined organic layers were dried over anhydrous sodium sulfate, and concentrated in vacuo. To the above residue and biotin-TFP[ii] (105 mg, 0.268 mmol) in DMF (4.9 mL) was added TEA (73 μL, 0.524 mmol). After stirred at room temperature for 12 h, the reaction mixture was concentrated in vacuo directly. The residue was purified by flash chromatography (silica gel, DCM:MeOH = 20:1) to afford crude **Probe** (160 mg), which was further purified by prepared HPLC [35% H_2O-65% MeOH→20% H_2O -80% MeOH (10 min), (15 mL/min)] to afford pure **Probe** as a white foam (84.5 mg, 69%).

Positive probe (Probe): TLC (CH_2Cl_2:MeOH, 10:1 v/v): R_f = 0.42; $[\alpha]^{25}_D$ +16.0 (c 0.05, CH_2Cl_2), ^{1}H NMR (400 MHz, $CDCl_3$) δ 1.38-1.56 (m, 4H), 1.62-2.38 (m, 21H), 2.57-2.78 (m, 6H), 2.80-2.87 (m, 1H), 2.88-2.94 (m, 1H), 3.12-3.17 (m, 1H), 3.20-3.28 (m, 2H), 3.44 (q, *J* = 5.2 Hz, 2H), 3.55-3.67 (m, 10H), 3.77-3.80 (m, 3H), 3.98 (s, *br*, 1H), 4.10 (dd, *J* = 9.2 Hz, 11.2 Hz, 1H), 4.28-4.33 (m, 2H), 4.49-4.52 (m, 1H), 4.85 (s, 1H), 4.97 (s, 1H), 4.98 (s, 1H), 4.99 (s, 1H), 5.10 (s, *br*, 1H), 5.48 (d, *J* = 3.2 Hz, 2H), 5.63 (t, *J* = 6.0 Hz, 1H), 5.86 (s, *br*, 1H), 6.17 (d, *J* = 3.6 Hz, 1H), 6.19 (d, *J* = 3.6 Hz, 1H), 6.79 (s, *br*, 1H); ^{13}C NMR (100 MHz, $CDCl_3$) δ 24.4, 25.5, 28.0, 28.1, 29.3, 29.7, 30.9, 32.0, 35.6, 35.9, 37.0, 37.5, 38.2, 39.1, 40.5, 42.5, 45.3, 45.7, 48.0, 53.7, 55.0, 55.4, 60.1, 60.3, 61.8, 66.7, 69.9, 70.1, 70.4, 70.4, 76.0, 76.7, 83.0, 83.5, 87.8, 90.5, 113.2, 115.1, 120.1, 120.2, 139.3, 139.6, 147.3, 147.6, 163.5, 169.9, 170.2, 171.8, 173.3; IR (neat) ν_{max} 3334, 3054, 2985, 2930, 2869, 1762, 1703, 1526, 1462, 1266, 1144, 994, 908, 745 cm^{-1}; HRMS (ESI) [M + H^+] calculated for $C_{49}H_{68}N_3O_{13}S$: 938.4467, found: 938.4454.

Formation of inactive analogue 3 from 2

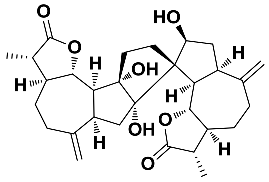

13: The freshly prepared solution of $NaBH_4$ (10.4 mg, 0.276 mmol) in MeOH (0.5 mL) was added to the solution of **2** (70.0 mg, 0.138 mmol) in MeOH (6.0 mL) and CH_2Cl_2 (1.5 mL) for seven times (total amount of $NaBH_4$: 72.8 mg, 1.932 mmol) over 3 h at 0 ^{o}C. The reaction mixture was quenched by acetone (5 mL) before poured into sat. aqueous NH_4Cl (50 mL). The resulting mixture was extracted with EtOAc (50 mL×3). The combined organic layers were washed with brine, dried over anhydrous sodium sulfate, and concentrated in vacuo. The residue was purified by flash chromatography (silica gel, EtOAc:PE = 2:1) to afford **13** (67 mg, 95 %) as a white foam.

13: TLC (EtOAc:PE, 4:1 v/v): R_f = 0.41; $[\alpha]^{21}_D$ +19.0 (*c* 0.20, CH_2Cl_2); ^{1}H NMR (400 MHz, $CDCl_3$) δ 1.20 (d, *J* = 7.2 Hz, 3H), 1.22 (d, *J* = 6.8 Hz, 3H), 1.26-1.35 (m, 3H), 1.64-1.67 (m, 1H), 1.68-2.15 (m, 11H), 2.18-2.30 (m, 3H), 2.44-2.78 (m, 6H), 2.83 (s, *br*, 1H), 3.23 (m, 1H), 4.09 (dd, *J* = 9.2 Hz, 10.8 Hz, 1H), 4.15 (dd, *J* = 9.6 Hz, 11.2 Hz, 1H), 4.38 (dd, *J* = 5.2 Hz, 11.6 Hz, 1H), 4.86 (s, 1H), 4.91 (s, 1H), 5.01 (s, 1H), 5.23 (s, 1H); ^{13}C NMR (100 MHz, $CDCl_3$) δ 13.0, 13.3, 21.8, 32.6, 32.7, 34.1, 36.4, 38.3, 38.7, 41.6, 41.7, 42.5, 43.2, 50.3, 52.5, 53.1, 53.3, 60.0, 71.7, 81.9, 83.9, 88.8, 90.6, 113.1, 113.3, 147.5, 148.4, 177.9, 178.0; IR (neat) ν_{max} 3379, 3055, 2929, 2957, 1770, 1455, 1380, 1338, 1266, 1235, 1213, 987, 743 cm^{-1}; HRMS (ESI) [M + Na^+] calculated for $C_{30}H_{40}NaO_7$: 535.2666, found: 535.2668.

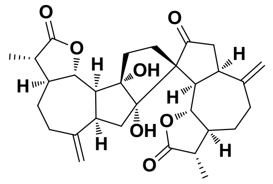

3: To a solution of oxalyl chloride (2.5 μL, 0.029 mmol) in CH_2Cl_2 (0.1 mL) was added the solution of DMSO (2.5 μL, 0.035 mmol) in CH_2Cl_2 (0.1 mL) at -78 ^oC. After 1.5 h, a solution of **13** (3.0 mg, 0.0059 mmol) in CH_2Cl_2 was added. The reaction mixture was stirred at the same temperature for 1.5 h before Et_3N (8.5 μL, 0.061 mmol) was added. The resulting mixture was stirred for another 1.5 h at -78 ^oC and 0.5 h at room temperature. The reaction mixture was poured into brine (15 mL), and extracted with EtOAc (15 mL×3). The combined organic layers were dried over anhydrous sodium sulfate, and concentrated in vacuo. The residue was purified by flash chromatography (silica gel, EtOAc:PE = 11:9) to afford **3** (1.7 mg, 57 %) as a foam, along with the recovered **13** (1.2 mg, 40%).

3: TLC (EtOAc:PE, 4:1 v/v): R_f = 0.55; $[\alpha]^{21}_D$ +62.0 (*c* 0.65, CH_2Cl_2); ^1H NMR (400 MHz, $CDCl_3$) δ 1.21 (d, *J* = 6.8 Hz, 3H), 1.25 (m, 1H), 1.28 (d, *J* = 6.8 Hz, 3H), 1.37-1.42 (m, 1H), 1.78-1.82 (m, 2H), 1.91 (dd, *J* = 3.6 Hz, 14.4 Hz, 1H), 1.98-2.02 (m, 2H), 2.07-2.32 (m, 9H), 2.39-2.43 (m, 2H), 2.50-2.55 (m, 1H), 2.60-2.65 (m, 1H), 2.85-2.89 (m, 1H), 2.94 (t, *J* = 10.0 Hz, 1H), 3.00-3.17 (m, 3H), 3.74 (s, *br*, 1H), 4.00-4.08 (m, 2H), 4.58 (s, 1H), 4.92 (s, 1H) , 4.98 (s, 1H), 5.11 (s, 1H); ^{13}C NMR (100 MHz, $CDCl_3$) δ 13.1, 13.7, 26.2, 32.8, 33.0, 33.2, 37.9, 38.2, 38.8, 39.1, 41.9, 41.9, 46.8, 48.2, 50.5, 53.1, 53.1, 62.2, 82.7, 83.7, 89.4, 90.2, 113.0, 113.3, 148.0, 150.3, 177.3, 178.3, 224.9; IR (neat) ν_{max} 3453, 2974, 2933, 2857, 1779, 1718, 1639, 1457, 1381, 1341, 1317, 1257, 1214, 991, 735 cm^{-1}; HRMS (ESI) [M + Na^+] calculated for $C_{30}H_{38}NaO_7$: 533.2510, found: 533.2510.

Preparation of negative probe (NC) from 13

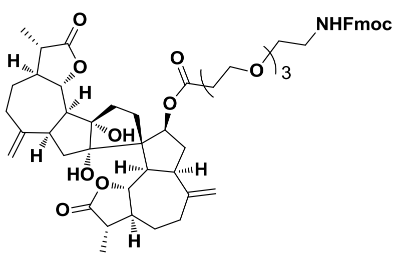

14: To a solution of Fmoc-12-amino-4, 7, 10-trioxadodecanoic acid (140 mg, 0.316 mmol) in toluene (3.2 mL) was added 2, 4, 6-trichlorobenzoyl chloride (246 μL, 1.52 mmol) and DIPEA (332 μL, 1.90 mmol). After stirred at room temperature for 45 min, the reaction mixture was concentrated in vacuo. The residue was dissolved in benzene (12 mL) and added to **13** (65 mg, 0.127 mmol). To the mixture was added the solution of DMAP (186 mg, 1.52 mmol) in benzene (6.0 mL) over 20 min. The reaction mixture was stirred at room temperature for 3 h before poured into the mixture of 0.5 M aqueous HCl (30 mL) and EtOAc (10 mL). The organic layer was separated and the aqueous layer was extracted with EtOAc (30 mL×2). The combined organic layers were washed with sat. aqueous $NaHCO_3$, brine, dried over anhydrous sodium

sulfate, and concentrated in vacuo. The residue was purified by flash chromatography (silica gel, EtOAc:PE = 2:1) to afford **14** (81 mg, 68 %) as colorless oil.

14: TLC (EtOAc:PE, 1:1 v/v): R_f = 0.55; $[\alpha]^{23}_D$ +12.3 (*c* 0.20, CH_2Cl_2); ^{1}H NMR (400 MHz, $CDCl_3$) δ 1.20 (d, *J* = 7.2 Hz, 3H), 1.20 (d, *J* = 6.8 Hz, 3H), 1.28-1.40 (m, 2H), 1.66-1.87 (m, 7H), 1.93-2.47 (m, 13H), 2.58-2.61 (m, 3H), 3.14-3.19 (m, 2H), 3.30 (s, *br*, 1H), 3.39-3.42 (m, 2H), 3.52-3.63 (m, 10H), 3.72-3.79 (m, 2H), 4.05 (dd, *J* = 10.0 Hz, 11.2 Hz, 1 H), 4.13-4.25 (m, 2H), 4.39 (d, *J* = 7.2 Hz, 2H), 4.83 (s, 1H), 4.91 (s, 1H), 4.94 (s, 1H), 4.96 (m, 1H), 5.55 (s, *br*, 1H), 5.61 (t, *J* = 6.2 Hz, 1H), 7.31 (t, *J* = 6.8 Hz, 2H), 7.39 (t, *J* = 7.6 Hz, 2H), 7.61 (d, *J* = 7.6 Hz, 2H), 7.76 (d, *J* = 7.6 Hz, 2H); ^{13}C NMR (100 MHz, $CDCl_3$) δ 13.0, 13.1, 24.0, 29.7, 30.2, 31.6, 32.1, 32.6, 35.6, 36.9, 37.8, 38.1, 40.9, 41.6, 41.7, 42.2, 44.9, 47.3, 50.3, 52.6, 53.0, 54.1, 60.2, 66.6, 66.6, 70.1, 70.3, 70.4, 70.5, 70.5, 75.7, 83.4, 83.5, 88.4, 90.5, 112.4, 114.4, 119.9, 125.1, 127.0, 127.6, 141.3, 144.0, 147.7, 148.2, 156.6, 171.8, 178.3, 178.4; IR (neat) ν_{max} 3439, 3055, 2934, 1770, 1723, 1675, 1639, 1517, 1451, 1422, 1350, 1267, 1182, 1117, 986, 734, 704 cm^{-1}; HRMS (ESI) [M + Na^+] calculated for $C_{54}H_{67}NNaO_{13}$: 960.4505, found: 960.4485.

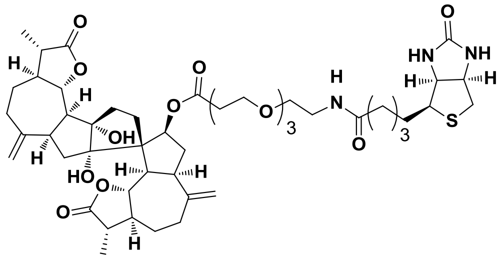

Negative probe (NC): To a solution of **14** (60 mg, 0.064 mmol) in DMF/*i*-PrOH (10:1, 1.2 mL) was added TBAF (2.5 mL, 0.100 mmol, 0.04 M in DMF). The reaction mixture was stirred at room temperature for 1 h before pouring into the mixture of EtOAc (25 mL) and brine (25 mL). The aqueous layer was separated, and the organic layer was washed with brine (25 mL×2). The aqueous layers were further extracted with EtOAc (25 mL) sequentially. The combined organic layers were dried over anhydrous sodium sulfate, and concentrated in vacuo. The above residue and biotin-TFP (52.5 mg, 0.134 mmol) was dissolved in DMF (2.5 mL), and to the mixture was added TEA (37 μL, 0.26 mmol). After stirred at room temperature for 12 h, the reaction mixture was concentrated in vacuo directly. The residue was purified by flash chromatography (silica gel, DCM:MeOH = 30:1) to afford the crude **NC** (60 mg), which was further purified by prepared HPLC [35% H_2O-65% MeOH→100% MeOH (8 min), (15 mL/min)] to afford pure **NC** as a colorless oil (30.0 mg, 50%).

Negative probe (NC): TLC (DCM:MeOH, 10:1 v/v): R_f = 0.35; $[\alpha]^{20}_D$ +30.0 (c 0.11, CH_2Cl_2); ^{1}H NMR (400 MHz, $CDCl_3$) δ 1.21-1.25 (m, 6H), 1.24-1.30 (m, 1H), 1.38-1.50 (m, 4H), 1.67-1.89 (m, 9H), 2.06-2.49 (m, 14H), 2.59-2.75 (m, 4H), 2.92 (dd, *J* = 5.2 Hz, 12.8 Hz, 1H), 3.16-3.38 (m, 4H), 3.44-3.48 (m, 2H), 3.56 (m, 2H), 3.62-3.84 (m, 12H), 4.08 (t, *J* = 10.4 Hz, 1H), 4.16 (t, *J* = 10.0 Hz, 1H), 4.31 (m, 1H), 4.51 (m, 1H), 4.84 (s, 1H),

4.93-4.97 (m, 4H), 5.54 (s, 1H), 5.62 (t, *J* = 6.4 Hz, 1H), 6.57 (t, *J* = 5.6 Hz, 1H); ^{13}C NMR (100 MHz, $CDCl_3$) δ 13.1, 13.2, 13.7, 19.7, 24.0, 24.2, 25.5, 28.1, 30.3, 31.7, 32.1, 32.6, 35.6, 35.9, 37.0, 37.8, 38.0, 39.1, 40.6, 41.7, 41.8, 42.4, 43.6, 44.9, 50.3, 52.5, 53.0, 54.3, 55.3, 60.1, 60.2, 61.7, 66.7, 69.8, 70.1, 70.4, 76.0, 83.6, 83.6, 88.3, 90.5, 112.4, 114.4, 147.9, 148.3, 163.1, 171.8, 173.1, 178.5, 178.7; IR (neat) ν_{max} 3343, 2932, 2872, 1769, 1699, 1548, 1456, 1266, 1183, 1119, 1013, 985, 734 cm^{-1}; HRMS (ESI) [M + Na^+] calculated for $C_{49}H_{71}N_3NaO_{13}S$: 964.4600, found: 964.4580.

Supplementary References

[1] S. Keil, C. Claus,W. Dippold, H. Kunz. *Angew. Chem. Int. Ed*. **2001**, *40*, 366.

[2] Wilbur D. S., Hamlin D. K., Pathare P. M., Weerawarna S. A. *Bioconjugate Chem*. **1997,** *8*, 572.
